# Evaluation of a flavonoids library for inhibition of pancreatic α-amylase towards a structure–activity relationship

**DOI:** 10.1080/14756366.2018.1558221

**Published:** 2019-02-06

**Authors:** Carina Proença, Marisa Freitas, Daniela Ribeiro, Sara M. Tomé, Eduardo F. T. Oliveira, Matilde F. Viegas, Alberto N. Araújo, Maria J. Ramos, Artur M. S. Silva, Pedro A. Fernandes, Eduarda Fernandes

**Affiliations:** a LAQV, REQUIMTE, Laboratory of Applied Chemistry, Department of Chemical Sciences, Faculty of Pharmacy, University of Porto, Porto, Portugal;; b Department of Chemistry and QOPNA, University of Aveiro, Aveiro, Portugal;; c UCIBIO, REQUIMTE, Department of Chemistry and Biochemistry, Faculty of Sciences, University of Porto, Porto, Portugal

**Keywords:** α-Amylase inhibition, diabetes, docking, flavonoids, *in vitro*

## Abstract

α-Amylase has been considered an important therapeutic target for the management of type 2 diabetes *mellitus* (T2DM), decreasing postprandial hyperglycaemia (PPHG). In the present work, a panel of 40 structurally related flavonoids was tested, concerning their ability to inhibit α-amylase activity, using a microanalysis screening system, an inhibitory kinetic analysis and molecular docking calculations. From the obtained results, it was possible to observe that the flavone with a -Cl ion at 3-position of C-ring, an –OH group at 3′- and 4′- positions of B-ring and at 5- and 7- positions of A-ring and the C2 = C3 double bond, was the most active tested flavonoid, through competitive inhibition. In conclusion, some of the tested flavonoids have shown promising inhibition of α-amylase and may be considered as possible alternatives to the modulation of T2DM.

## Graphical Abstract

**Figure:**
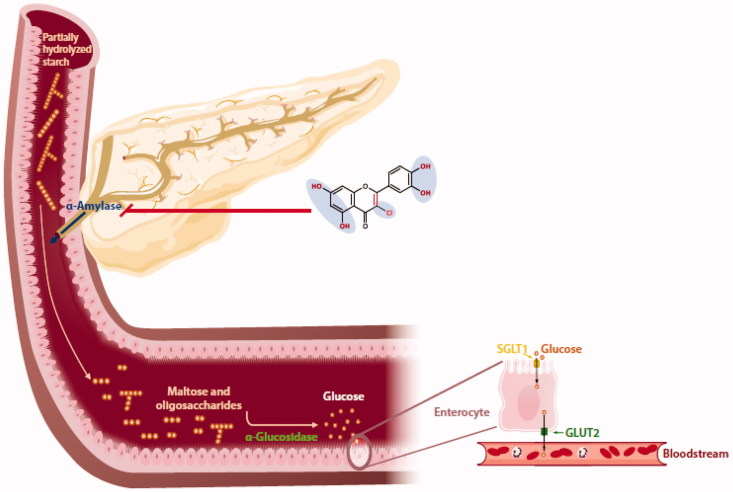

## Introduction

α-1,4-Glucan-4-glucanohydrolases (EC 3.2.1.1.), belonging to the family 13 of glycoside hydrolases (GH) and found in saliva and pancreatic juice, are known by the common name of α-amylase^1^ ^,^ ^2^. Both are isozymes expressed respectively from genes AMY1 and AMY2 and are composed of 496 amino acid residues in a single polypeptide chain, presenting a 97% identity of the amino acids sequence and a 92% similarity of the residues in the catalytic domains^3–5^. They have three different domains (A, B and C) and a common calcium-binding site that stabilizes the interface between the central A domain and the variable B domain^6^ ^,^ ^7^. The catalytic triad (Asp, Asp, Glu) is present in the A domain and the active site is located in a long cleft between the carboxyl end of both A and B domains. The C domain consists of a β-sheet structure linked to the A domain by a simple polypeptide chain^5^.

**Figure 1.**
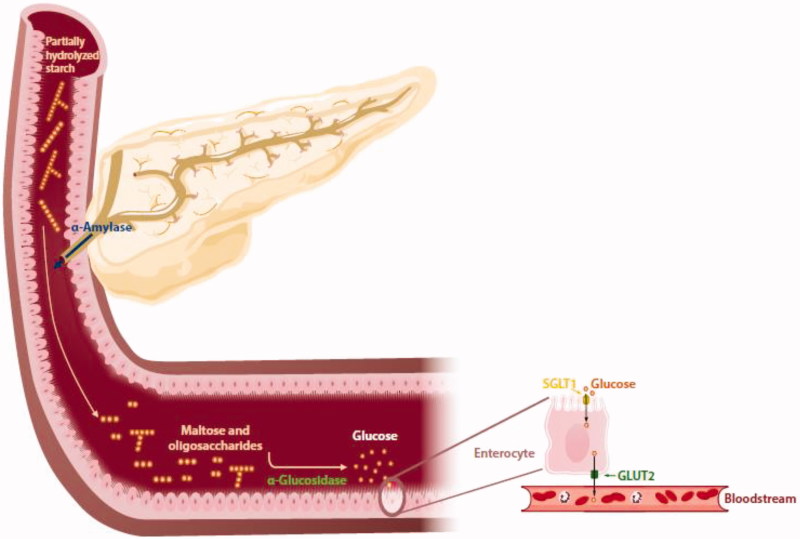
Schematic representation of the pancreatic α-amylase and α-glucosidase activity. After a meal, α-amylase synthesized in pancreas and released in the duodenum, catalyzes the hydrolysis of α-1,4 glycosidic linkages in partially hydrolyzed starch (amylopectin and amylose). From this reaction, intermediate unbranched, such as maltose and maltotriose, and branched (α-limit dextrins) oligosaccharides are formed. α-Glucosidase present in the brush border of the intestinal epithelium (enterocytes) is responsible for the final step of carbohydrates digestion, prior to their absorption. This enzyme converts the disaccharides and oligosaccharides into glucose, which is then transported by sodium/glucose co-transporter 1 (SGLT1) from the intestinal lumen to the cytosol of enterocytes. In turn, glucose transporter 2 (GLUT2), found in the basolateral membrane of enterocytes, transports glucose from cytosol to blood via facilitated diffusion.

α-Amylase catalyzes the initial hydrolysis of starch and other carbohydrate polymers into shorter oligosaccharides through cleavage of α-1,4- bonds^5^ ^,^ ^7^ ^,^ ^8^. The salivary isozyme provides an initial partial cleavage into shorter oligomers (10–30%)^7^. Once these partially digested saccharides reach the gut, they are extensively hydrolyzed by the α-amylase synthesized in the pancreas and excreted in the lumen into smaller oligosaccharides, such as maltose, maltotriose and α-limit dextrins (Figure 1)^3^. These sugars are finally broken down into glucose by the intestinal brush border α-glucosidases, which is in turn absorbed from the intestinal mucosa into the portal blood, by means of the glucose transporter (GLUT2) and sodium-glucose co-transporter 1 (SGLT1), leading to postprandial hyperglycaemia (PPHG)^2^. Impaired regulation of PPHG constitutes a common feature in type 2 diabetes *mellitus* (T2DM), the most prevalent form of diabetes, accounting for about 90% of all diabetes cases^2^ ^,^ ^9^ ^,^ ^10^.

The pancreatic α-amylase activity has been targeted for inhibition by means of the so-called starch blockers in order to mitigate PPHG^1^ ^,^ ^3^. Acarbose is the most widely prescribed α-amylase inhibitor, and in spite of its efficiency in the control of PPHG, the administration of this drug is associated with gastrointestinal adverse effects in diabetic patients, namely abdominal distention, flatulence and diarrhoea^11^. Thus, the search and development of new effective and safer agents, able to control glucose levels is of high importance for the management of T2DM^12^.

**Figure 2.**
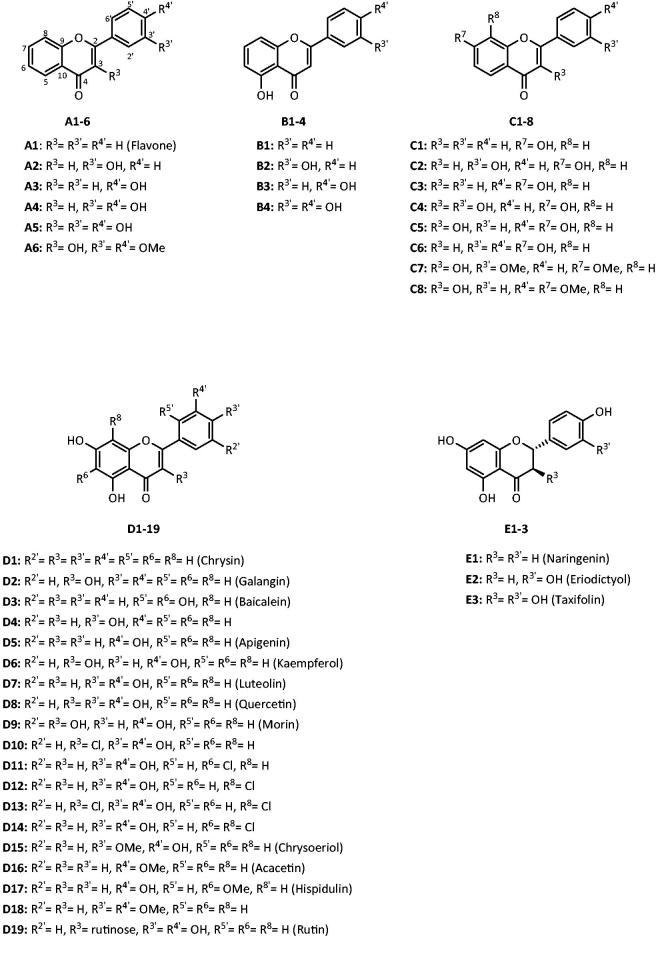
Chemical structures of the studied flavonoids.

In the last few years, the activity of flavonoids has been studied even up to clinical trials concerning the modulation of diabetes in humans^13^ ^,^ ^14^. Promising inhibitory potential against important targets related with the T2DM pathophysiology was evidenced regarding the inhibition of isolated enzymes such as α-glucosidase^15^ and protein tyrosine phosphatase 1B (PTP1B)^16^. Some bibliographic reviews compile the obtained results of the antidiabetic activity of flavonoids^17–20^. Further isolated studies also describe flavonoids as α-amylase inhibitors, such as apigenin^21^ ^,^ ^22^, luteolin^21^ ^,^ ^23^, kaempferol^21^ ^,^ ^22^, naringenin^21^, quercetin^21^ ^,^ ^22^ ^,^ ^24^ ^,^ ^25^, myricetin^21^ ^,^ ^22^ ^,^ ^25^, chrysin^22^ and baicalein^21^ ^,^ ^22^. Nevertheless, significant differences in the experimental conditions among studies, such as the source and concentrations of enzyme and substrate and the different incubation times applied, turn difficult the establishment of a reliable structure-activity relationship. To fill this gap, a panel of 40 structurally related flavonoids (Figure 2), most of them studied for the first time, was assessed regarding their inhibitory activity against α-amylase. This study comprehends an *in vitro* microanalysis screening system, modelling of kinetics inhibition and an *in silico* molecular docking analysis.

## Materials and methods

### Chemicals

α-Amylase from porcine pancreas, 2-chloro-4-nitrophenyl-α-D-maltotrioside (CNPG3), **acarbose**, DMSO, NaHPO_4_, Na_2_HPO_4_, flavonoids **D3** (baicalein), **D4** (apigenin), **D6** (kaempferol), **D7** (luteolin), **D8** (quercetin), **D9** (myricetin), **D10** (morin), **D17** (acacetin), **D19** (rutin), **E1** (naringenin), **E2** (eriodictyol) and **E3** (taxifolin) were obtained from Sigma-Aldrich Co. LLC (St. Louis, MO). The following flavonoids were obtained from Indofine Chemical Company, Inc. (Hillsborough, NJ): **A1**, **A2**, **A3**, **A4**, **A5**, **A6**, **B1**, **B2**, **B3**, **B4**, **C1**, **C2**, **C3**, **C4**, **C5**, **C6**, **C7**, **C8**, **D1** (chrysin), **D2** (galangin) and **D5**. The flavonoids **D11**, **D12**, **D13**, **D14**, **D15, D16**and **D18** were synthesized according to previous companion papers^26^ ^,^ ^27^.

### *In vitro* pancreatic α-amylase inhibition assay

The evaluation of inhibitory effect on α-amylase activity was based on the method described by Trinh et al.^28^, with slight modifications. In each assay, the α-amylase mediated hydrolysis of the substrate CNPG3 into 2-chloro-nitrophenol (CNP), 2-chloro-4-nitrophenyl-α-D-maltoside (CNPG2), maltotriose and glucose was monitored. The initial rate of CNP generation, measured spectrophotometrically at 405 nm, is proportional to the concentration of α-amylase present. In brief, in a 96-well plate, the enzyme (0.2 U/mL), dissolved in 20 mM phosphate buffer (20 mM Na_2_HPO_4_ and 7 mM NaCl, pH 6.8) was exposed to the flavonoids under study (0–200 µM), dissolved in DMSO [final concentration of DMSO of 4.5% (*v/v*)]. After an incubation time of 10 min, at 37 °C, the reaction was started by the addition of CNPG3 (0.5 mM), dissolved in 20 mM phosphate buffer (pH 6.8) and monitored along 30 min, at 37 °C, in a microplate reader (Synergy HT, BIO-TEK), with the wavelength set to 405 nm. The concentration of α-amylase and the tested flavonoids was determined before the addition of the substrate. Increasing absorbance values obtained within the 5 to 30 min interval (slope) served for the calculation of catalytic rates and the results in the form of inhibition percentages, representing at least three independent experiments. **Acarbose** (0–16 µM) was used as positive control. The amount of DMSO used did not interfere with the assay. The results of the *in vitro* inhibitory activity of flavonoids against the pancreatic α-amylase activity are expressed as mean ± standard error of mean (SEM). Statistical comparison between the active flavonoids was estimated by applying the one-way analysis of variance (ANOVA). Differences were considered to be significant at *p* values lower than 0.05. All the statistical analysis were performed using GraphPad Prism™ (version 7.0; GraphPad Software).

### Inhibitory kinetics of α-amylase

The estimation of kinetic parameters and prediction of the actual mechanism of inhibition was performed by means of Microsoft Office Excel™ spreadsheets and using the Solver supplement add-in, according to Bezerra et al.^29^ and Dias et al.^30^. Therefore, the kinetics of conversion of CNPG3 by α-amylase in each microplate well was fitted by nonlinear least squares regression^31^ using the general model for enzymatic reactions translated by the Equation (1), and each one of its simplifications regarding the different types of inhibition:

(1) $$v_{\text{inic}}=\frac{V_{máx}(S)}{K_{m}(1+\frac{\left|I\right|}{K_{\text{ic}}})+(S)(1+\frac{\left|I\right|}{K_{\text{iu}}})}$$

where *v*_inic_ and *V*_max_ represent respectively the initial velocity of formation of absorbing CNP in µmol/min and the maximum achievable velocity when for the 0.2 U/mL of enzyme used all catalytic sites are saturated by the substrate, S is the CNPG3 concentration in mM, *K*_m_ is the Michaelis–Menten constant in mM and *K*_ic_, *K*_iu_ are the constants for competitive and uncompetitive inhibition processes expressed in µM^−1^.

To study the enzyme kinetics, nonlinear regression was applied to the nontransformed data for the most active flavonoids of each group: **B4** (0–200 µM), **C5** (0–100 µM), **D11** (0–100 µM) and **acarbose** (0–2 µM). For each concentration of inhibitor, three concentrations of the substrate CNPG3 were tested: 0.25, 0.5 and 1 mM. The obtained results represent at least three independent experiments.

For each tested condition, the best guesses for equation parameters were thrown by Solver tool after iterative minimization of the sum of squared residuals between the experimental values of *V*_max_ and the corresponding counterparts estimated according to each model used. Hence, the values obtained for the parameters of the simplest model (without inhibition) were used as initial values, proceeding to competitive inhibition, noncompetitive inhibition, uncompetitive inhibition and finishing with the more complex model, mixed inhibition. The actual mechanism of inhibition was established after comparison and discrimination between the models with a different number of parameters applying the extra sum-of-square *F* test^32^ and the Akaike information criterion (AIC) test^33^.

The jackknife procedure was applied to determinate the error of the kinetic constant values. It consisted of the calculation of standard deviation of all estimates guessed by Solver when each experimental data point was in turn drawn from the initial set^34^.

### Molecular docking analysis

Seventeen entries in the Protein Data Bank (PDB) correspond to *Sus Scrofa* α-amylase (E.C. 3.2.1.1.). From these, it was chosen the PDB entry 1HX0^35^ corresponding to the highest resolution X-ray structure (1.38). This structure was co-crystalized with a trisaccharide inhibitor K2, derived from acarbose after α-amylase cleaved one glucose unit at the reducing end. The crystal structure shown two α-1,4-linked K2 molecules, condensed *in situ*. The K2 unit bound at the active site was used to compare the binding mode of the tested flavonoids and as a template to model the substrate for receptor minimization and conformational sampling.

The protonation states of the enzyme were predicted using the Protoss server^36^. Accordingly, Glu233 and Glu390 were modelled in the neutral form and His101, His201 and His215 were modelled in the positively charged for, with both side chain nitrogen atoms protonated. All other residues were protonated according to their p*K*a, at pH = 7.

From the hexasaccharide compound present in 1HXO (resultant of two α-1,4-linked K2 molecules), the substrate maltohexose was modelled. On this system, Molecular Mechanics minimization was performed, followed by 20 ns of classical molecular dynamics (MD) simulation to sample receptor conformations. This protocol was performed within the AMBER 12 suite^37^ using the ff99SB^38^ force field for the protein and GLYCAM06^39^ force field for maltohexose. Further details for the energy minimization and MD simulation can be found in Supporting Information.

To choose and validate the conformation of the receptor for the docking protocol several receptor conformations were evaluated: the X-ray conformation, the conformation of the receptor minimized with maltohexose and four representative conformations of the clustered MD simulation. The suitability of each conformation was assessed by evaluating if the docking of the inhibitor acarbose in the active site reproduced the X-ray conformation and how well the ranking of the tested flavonoids with the lowest IC_50_ was reproduced. The conformation of the receptor with the best performance was obtained after 3.3 ns of simulation.

The binding mode of the selected flavonoids was predicted by means of the AutoDock Vina^40^. The binding poses were generated using default parameters (see Supporting Information for details), such as the exhaustiveness of the global search (8) or the maximum number of output poses (9). The search space was confined to a box of 19 Å × 12 Å × 17 Å centered on the site occupied by the glycosidic oxygen between the –1 and –2 glucose units of maltohexose. The receptor was treated as a rigid body and prepared using openbabel^41^ for atom-typing and charge assignment using Gasteiger–Marsili charges^42^. The atom-types and charges for the tested flavonoids were attributed the same way.

Subsequently, the poses predicted by AutoDock Vina for each flavonoid were scored using the scoring function GoldScore within the GOLD software suite^43^, using the standard parameters for a “Rescoring Run” with local optimization.

## Results

### In vitro *pancreatic α-amylase inhibition assay*

The tested flavonoids were divided into five groups (A–E), according to their substitution pattern. The substitution pattern of flavonoids from the A group differs at 3-position of the C-ring and at 3′- and 4′-positions of the B-ring; flavones from B group have an –OH group at 5-position of the A-ring and different substitutions at 3′- and 4′-positions of the B-ring; flavonoids from C group show –OH, –OMe and/or –OBn groups at 3-position of the C-ring, at 3′- and 4′-positions of the B-ring and at 7- and 8-position of the A-ring; flavonoids from D group present –OH substitutions at 5- and 7- positions of the A-ring and also present –OH, –OMe and/or –Cl substitutions at 3-position of the C-ring, at 2′-, 3′-, 4′- and 5′-positions of the B-ring and at 6- and 8-positions of the A-ring; flavanones from E group have –OH groups at 5- and 7-positions of the A-ring and their substitution pattern differs in the presence or absence of –OH groups at 3′- and 4′-positions of the B-ring (Table 1).

**Table 1.** Inhibitory activity of flavonoids against porcine pancreatic α-amylase (IC_50_, µM ± SEM).

Compound	Structure	*R*^2^′	*R*^3^	*R*^3^′	*R*^4^′	*R*^5^′	*R*^6^	*R*^7^	*R*^8^	IC_50_ (µM) or Inhibition (%)
A1 (Flavone)		–	H	H	H	–	–	–	–	<20%^200^ µM*
A2		–	H	OH	H	–	–	–	–	<20%^200^ µM*
A3		–	H	H	OH	–	–	–	–	<20%^200^ µM*
A4		–	H	OH	OH	–	–	–	–	46 ± 2%^200^ µM*
A5		–	OH	OH	OH	–	–	–	–	31 ± 3%^200 ^µM*
A6		–	OH	OMe	OMe	–	–	–	–	<20%^200^ µM*
B1		–	–	H	H	–	–	–	–	<2%^200 ^µM*
B2		–	–	OH	H	–	–	–	–	<20%^200 ^µM*
B3		–	–	H	OH	–	–	–	–	<20%^200^ µM*
B4		–	–	OH	OH	–	–	–	–	148 ± 5 µM^(a,h)^
C1		–	H	H	H	–	–	OH	H	<20%^200^µM*
C2		–	H	OH	H	–	–	OH	H	28 ± 3%^200^µM*
C3		–	H	H	OH	–	–	OH	H	36 ± 3%^200^µM*
C4		–	OH	OH	H	–	–	OH	H	34 ± 3%^200^µM*
C5		–	OH	H	OH	–	–	OH	H	59 ± 4 µM^(b,c)^
C6		–	H	OH	OH	–	–	OH	H	131 ± 1 µM^(a,d)^
C7		–	OH	OMe	H	–	–	OMe	H	< 20 %^200^ µM*
C8		–	OH	H	OMe	–	–	OMe	H	< 20%^100 ^µM*
D1 (Chrysin)		H	H	H	H	H	H	–	H	22 ± 3%^200^µM*
D2 (Galangin)		H	OH	H	H	H	H	–	H	< 20%^200 ^µM*
D3 (Baicalein)		H	H	H	H	H	OH	–	H	< 20 %^200^µM*
D4		H	H	OH	H	H	H	–	H	≈ 200 µM^(e)^
D5 (Apigenin)		H	H	H	OH	H	H	–	H	122 ± 7 µM^(a,f)^
D6 (Kaempferol)		H	OH	H	OH	H	H	–	H	118 ± 7 µM^(a,f)^
D7 (Luteolin)		H	H	OH	OH	H	H	–	H	78 ± 3 µM^(b,g)^
D8 (Quercetin)		H	OH	OH	OH	H	H	–	H	138 ± 5 µM^(a)^
D9 (Myricetin)		H	OH	OH	OH	OH	H	–	H	107 ± 6 µM^(d,f)^
D10 (Morin)		OH	OH	H	OH	H	H	–	H	23 ± 1 %^200^µM*
D11		H	Cl	OH	OH	H	H	–	H	44 ± 3 µM^(c)^
D12		H	H	OH	OH	H	Cl	–	H	≈ 200 µM^(e)^
D13		H	H	OH	OH	H	H	–	Cl	173 ± 5 µM^(e,h)^
D14		H	Cl	OH	OH	H	H	–	Cl	82 ± 4 µM^(g)^
D15		H	H	OH	OH	H	Cl	–	Cl	98 ± 3 µM^(f)^
D16 (Chrysoeriol)		H	H	OMe	OH	H	H	–	H	192 ± 7 µM^(e)^
D17 (Acacetin)		H	H	H	OMe	H	H	–	H	<20%^200^ µM*
D18		H	H	OMe	OMe	H	H	–	H	33 ± 3 %^200^ µM*
D19 (Rutin)		H	Rutinose	OH	OH	H	H	–	H	<20%^200 ^µM*
E1 (Naringenin)		–	H	H	–	–	–	–	–	<20%^200 ^µM*
E2 (Eriodictyol)		–	H	OH	–	–	–	–	–	<20%^200 ^µM*
E3 (Taxifolin)		–	OH	OH	–	–	–	–	–	<20%^200 ^µM*
Positive control: Acarbose		–	–	–	–	–	–	–	–	1.3 ± 0.2 µM^(i^^)^

*Inhibition (mean, % ± SEM) at the highest tested concentration (in superscript), at the assay conditions.

The IC_50_ with different lowercase superscript letters are significantly different from each other (*p* < 0.05).

No relevant inhibitory activities were observed, up to the highest tested concentration (200 µM), for the flavones belonging to A group with different substitutions in 3-position of C-ring and in 3′- and 4′-positions of B-ring.

For flavones of B group, with an –OH group at 5-position of A-ring and different substitutions in 3′- and 4′-positions of B-ring, the most effective was **B4,** presenting an IC_50_ value of 148 ± 5 µM. This result demonstrates that the presence of the catechol group at B-ring translated in a relatively slight increase of the inhibitory effect over α-amylase catalysis when compared to the other flavones of this group. The presence of an –OH group at 5-position of A-ring also seems to contribute to such effect, since flavone **B4** was more active than flavone **A4** (also with 3′- and 4′–OH in B-ring, but without 5–OH in A-ring).

From C group, which includes flavonoids with different substitutions at 3-position of C-ring, at 3′ and 4′ positions of B-ring and at 7 and 8 positions of A-ring, the most active flavonoid was compound **C5**, tested here for the first time, with an IC_50_ value of 59 ± 4 µM. It seems that the presence of –OH groups at 3-position of C-ring, at 4′-position of B-ring and at 7-position of A-ring, is accompanied by a significant inhibitory effect on α-amylase. On the other hand, the presence of -OMe groups in the flavonoid scaffold (**C7**, **C8**) did not bring any advantage for the intended effect, since the initial conversion rates of the CNPG3 substrates remained unchanged even for the highest tested concentration (200 µM).

All members of D group have –OH groups at 5- and 7-positions of A-ring and the substitution pattern varies according to the presence or absence of –Cl ions, –OH and –OMe groups in their structure. The most effective flavonoid found was the chlorinated flavone **D11**, with an IC_50_ value of 44 ± 3 µM. Thus, the presence of a –Cl ion at 3-position of C-ring, as well as the presence of a catechol in B-ring and –OH groups at 5- and 7-positions of A-ring, showed to be crucial for the inhibition of α-amylase activity. Comparing flavonoid **D11** with **D8** (quercetin) (IC_50_= 138 ± 5 µM), it is clear that the substitution of the Cl- ion for an OH-group at 3-position of C-ring results in a significant decrease of the corresponding inhibitory activity. It was also observed that the presence of a -OMe group at 3′- position of B-ring [**D16** (chrysoeriol)] slightly improves the ability to inhibit α-amylase activity (IC_50_= 192 ± 7 µM), comparing with **D17** (acacetin), which presents a -OMe group at 4′-position of B-ring. An increase of the number of -OMe groups in the structure of flavonoids is not favourable for the intended effect, as it can be concluded from a comparison of **D16** (chrysoeriol) with **D18**, the later with an extra -OMe group at 4′-position of B-ring. A glycosylated flavonoid, **D19** (rutin), was also tested, but no activity was found up to the highest tested concentration (200 µM).

The studied flavanones of E group, **E1** (naringenin), **E2** (eriodictyol) and **E3** (taxifolin) were not able to inhibit α-amylase activity, at the maximum tested concentration (200 µM). These results demonstrate that the presence of C2 = C3 double bond is very relevant for the intended effect.

### Inhibitory kinetics of α-amylase

In accordance with the described results, the most effective flavonoids of B, C and D groups, **B4**, **C5** and **D11**, respectively, and the positive control, **acarbose**, were henceforth selected for their type of inhibition calculation. No flavonoids from A and E group were tested, due to their lack of inhibitory activity.

By comparing the sum of the square errors values among the different enzyme inhibition models and, for models with different number of parameters, applying the extra sum-of-square *F* test and the AIC test, it was possible to select the best model (without inhibition, competitive inhibition, noncompetitive inhibition, uncompetitive inhibition or mixed inhibition). In that sense, the model with the lowest sum of the square error value corresponds to the best kinetic model. The comparison between models with a different number of parameters can be done by comparing the extra sum-of-square *F* test, at the desired level of probability (ƒ 0.95): if the –ƒ value is superior to 0, the more complex model should be applied. The Akaike test was also used to discriminate the different enzyme inhibition models, with a different number of parameters: the model with the lower corrected AIC (AIC_c_) score is the model more likely to be correct.

By the obtained results, it was possible to conclude that **acarbose** [see Supporting Information, Figures 1(S)–5(S)] is a mixed-type inhibitor, and flavonoids **B4** [see Supporting Information, Figures 6(S)–10(S)], **C5** [see Supporting Information, Figures 11(S)–15(S)] and **D11** are competitive inhibitors [see Supporting Information, Figures 16(S)–20(S)].

After the definition of the best model, it was possible to summarize the kinetic constants values (*V*_max_, *K*_m_, *K*_ic_ and/or *K*_iu_) (Table 2). The tested compounds presented the following order of *K*_ic_ values: **B4**>**C5**>**D11**>**acarbose**.

**Table 2.** Type of inhibition and *V*_max_, *K*_m_, *K*_ic,_ and *K*_iu_values ± SD (µM) for the inhibition of α-amylase by the selected flavonoids.

Compound	Type of inhibition	*V*_max_(µmol/min)	*K*_m_ (mM)	*K*_ic_ (µM)	*K*_iu_ (µM)
**B4**	Competitive	12.0 ± 0.6	1.2 ± 0.1	132 ± 15	–
**C5**	Competitive	10.5 ± 0.9	1.0 ± 0.1	71 ± 7	–
**D11**	Competitive	15 ± 4	1.1 ± 0.4	21 ± 3	–
Positive control:					
**Acarbose**	Mixed	12.0 ± 0.9	0.9 ± 0.1	6 ± 3	0.71 ± 0.09

### Molecular docking analysis

#### Binding mode of the selected inhibitors

Once evidenced the inhibition mechanism through experimental data and calculations using Solver supplement of Microsoft Excel Office™, it was predicted the binding pose of flavonoids within the active site of α-amylase. Special attention is given to **B4**, **C5** and **D11** derivatives, the ones exhibiting a higher inhibitory effect in their groups.

The binding pocket of α-amylase is usually divided in –1 to N subpockets, each one creating a particular environment around each sugar ring. The innermost subpocket is numbered as –1 subpocket and is immediately followed the +1 subpocket while the glycosidic bond to be cleaved stays positioned in-between the sugars and also in-between the –1 and +1 subpockets. The next subpockets are numbered +2, +3, etc. An even deeper subpocket (–2) can be occupied by inhibitors, but generally not by natural substrates.

We started by evaluating the accuracy of our Molecular Docking Protocol. For this purpose, we performed a docking assay using the crystal structure of alpha-amylase complexed with acarbose (*Bacillus subtilis,* PDB structure 1UA7, and resolution 2.21 Å). We deleted the acarbose hexasaccharide from the 1UA7 PDB file and re-docked it into the active site, following the protocol described in the methods, using the same parameters, except for the size of the box (which was, in this case, 21.75 × 22.50 × 22.50 Å). The top-ranking solution of the docking assay displayed a very similar pose to that of acarbose in the crystallographic structure [see Supporting Information, Figure 21(S)]. Root mean square deviation (RMSD) analysis of the heavy atoms in the docking solution concurs with visual inspection, with a very low heavy-atom RMSD value of 0.482 Å, confirming that the used protocol has significant predictive power.

**Figure 3.**
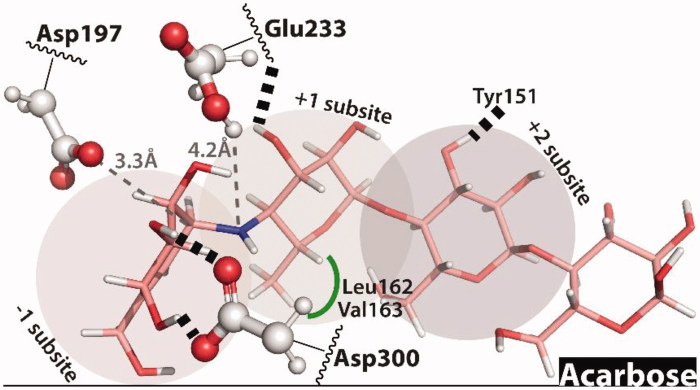
Predicted binding mode for **acarbose**. Hydrogen bonds are represented by dashed lines and hydrophobic interactions by green lines. The distances between the catalytic residues Asp197 and Glu233 and the anomeric carbon/N-glycosidic bond are represented by dashed gray lines.

The study started with the prediction of the binding mode of acarbose, which in the chosen receptor perfectly reproduces the binding interactions of the substrate maltohexose seen in the MD simulation (Figure 3). This was expected, as it was a condition that we imposed to choose the receptor conformation for the docking calculations. The hydroxymethyl conduritol ring of acarbose accommodates to the acarviosine moiety bound to the –1 and +1 subpockets while the two farthest glucose units extend from the +2 subpocket to onwards. In such conformation, this non-hydrolyzable *N*-glycosidic bond stays moderately distant from the general acid Glu233 (4.2 Å), but the nucleophilic Asp197 is only at 3.3 Å from the anomeric carbon, Asp300 is hydrogen bonded to the 2-hydroxyl and 3-hydroxyl groups of the glucose unit of the –1 subpocket. An additional hydrogen bond is established between the hydroxymethyl group and Asp197.

The most relevant interaction between the glucose unit in the +1 subpocket and the receptor seems to be the hydrophobic interaction between its methyl group and a hydrophobic pocket formed by Leu162 and Val163, and a hydrogen bond between the 3–OH and the backbone of Glu233. For the glucose unit at the +2 subpocket, the most relevant interaction seems to be a hydrogen bond between the 3–OH group and the side-chain of Tyr151. The glucose unit at the +3 subpocket is completely exposed to the solvent and does not interact meaningfully with the receptor.

Among flavonoids from A group, which carry substitutions at the 3-position of C-ring and at 3′- and 4′-positions of B-ring, a clear trend is observed: from flavonoids **A1** to **A5**, the –OH groups at 3-position of C-ring and at 3′-position of B-ring establish hydrogen bonds with Asp300, while the –OH group at 4′-position of B-ring establishes hydrogen bonds with Asp197, with this hydrogen bonding network being optimized in flavonoid **A5**.

Therefore, flavonoids **A1** to **A5** bind the active site with the B-ring oriented towards the catalytic triad (Asp197, Asp300, Glu233), occupying a site equivalent to the –1 subpocket of the K2 inhibitor in the PDB structure 1HXO^35^, while the A- and C-rings are pointing outwards occupying the binding site cleft equivalent to the –2 subpocket, which is a pocket not usually occupied by the natural substrate, located in the opposite direction in relation to the +1 subpocket.

For the less substituted **A1** to **A3** flavonoids, the described set of hydrogen bond interactions is not optimized and as such, they do not bind so deep in the active site. Instead, aromatic stacking interactions are observed between the A- and C-rings and Trp58, and between the B-ring and Tyr62.

**Figure 4.**
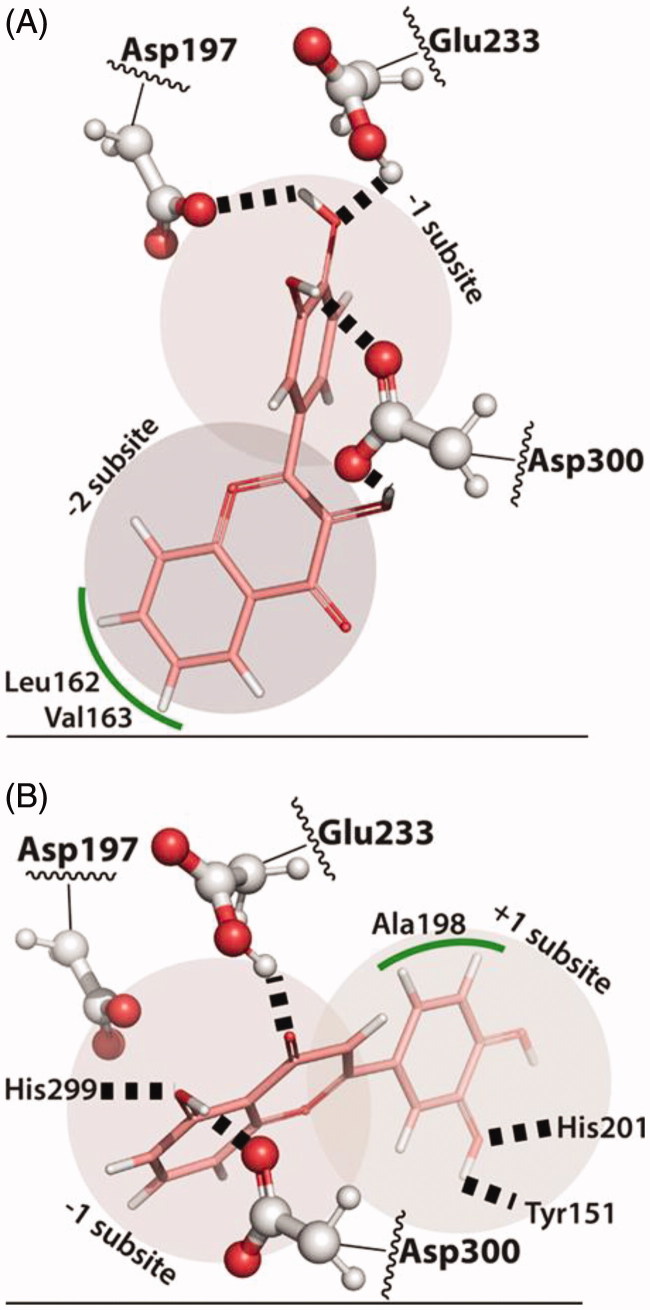
(A) Predicted binding mode of flavonoid **A5**, occupying the –2 and –1 subsites with the more hydrophobic A-ring on the –2 subsite interacting with Leu162 and Val163. (B) The predicted binding mode for **B4** shows the C-ring carbonyl and A-ring hydroxyl hydrogen bonded to the catalytic residues Glu233, Asp300 and His299.

When two –OH groups are added to B-ring (flavonoids **A4** and **A5**) the hydrogen bonds with the catalytic triad are optimized and these flavonoids bind deeper in the active site, hydrogen bonding with Asp197, Glu233 and Asp300, which might explain the higher inhibitory activity observed for these two flavones within A group [Figure 4(A)].

The steric hindrance introduced by methoxylation at 3′- and 4′-positions of B-ring (flavonoid **A6**), flips the binding pose, placing instead the A- and C-rings in the –1 subpocket. This allows the –OH group at 3-position of C-ring to establish a hydrogen bond with Asp300 and Glu233.

As evidenced before, within B group of structurally related flavonoids, the best inhibitor is **B4** with an IC_50_ value of 148 ± 5 µM. This compound shares with **A4** and **A5** the same hydroxyl substituents in the B-ring, but an additional –OH group at 5-position of the A-ring, which jointly promotes a stabilized conformation and an increase of the inhibition of α-amylase activity.

Looking at the pose predictions, it is clear that the C-ring carbonyl and the –OH group at the A-ring, promote the positioning of the A- and C-rings close to the catalytic triad. The orientation of the flavonoid, however, depends on the substitutions present in the B-ring [Figure 4(B)].

The two –OH substitutions present in the B-ring of flavonoid **B4** increase the hydrophilicity of the catechol, which seems to distinguish **B4** from the rest of the group. This allows the B-ring to occupy the +1 subpocket, with His201 hydrogen bonding the oxygen of the –OH group at 3′ position of B-ring, while that same –OH group makes a hydrogen bond with the oxygen of the Tyr151 hydroxyl [Figure 4(B)]. This orientation is unlike **B1** and **B2**, where the more hydrophobic B-ring binds the –2 subpockets, in the more hydrophobic region formed by Val163 and Leu165. Despite positioning the A- and C-ring between the catalytic triad, as the other flavonoids, this different orientation of the A- and C-ring of **B4**, seems to optimize the hydrogen-bonding network around the active site. Namely, the –OH group in the 5-position of A-ring establishes a hydrogen bond with Asp300 and to His299, while the C-ring carbonyl is hydrogen bonded to Glu233.

**Figure 5.**
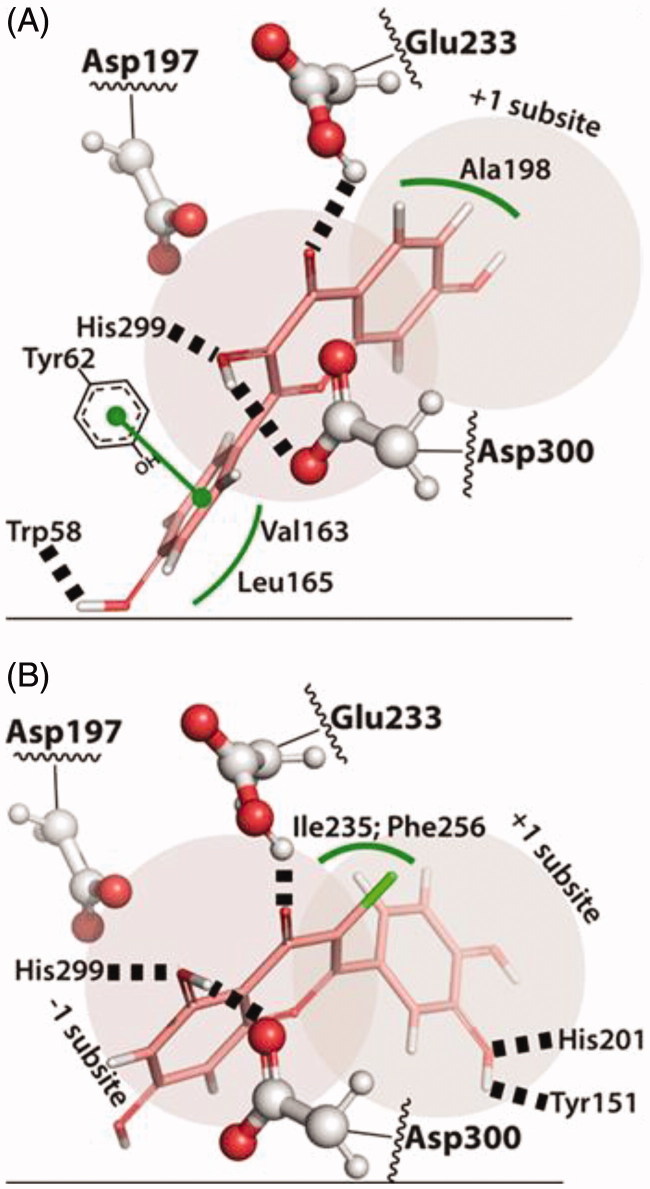
(A) The binding mode predicted for **C5** shows the C-ring carbonyl and –OH group interacting with the active site residues in a similar way as in **B4**. This requires the B-ring to be placed in the –1/–2 subsites, stabilized mostly by hydrophobic interactions with Tyr62, Val163 and Leu165. (B)The best tested inhibitor, flavonoid **D11**, and flavonoid **B4** binds very similarly to α-amylase. The most important difference seems to come from the 3–Cl substitution that interacts with Ile235 and Phe256.

In turn, from C group, the 3–OH substitution present in C-ring of flavonoids **C4** and **C5** gives rise to a substructural motif of adjoined hydroxyl and carbonyl groups, similarly to the one found in the B group. On the latter, a C-ring carbonyl group adjoined by a –OH group at 4-position of A-ring allows the positioning of A- and C-rings within the catalytic triad. Therefore, the C-ring carbonyl and 3–OH substitution in **C4** and **C5** flavonoids also promotes the placing of the A- and C-rings within the catalytic triad, with the hydrogen of the –OH group at 3-position of C-ring bonded to Asp300 and to His299, and with the Glu233 hydrogen bonding the carbonyl group [Figure 5(A)]. These positions of the carbonyl and –OH groups, exactly as in **B4,** and the same hydrogen-bonding network, are observed between these groups and the active site. This also means that **C4** and **C5** are bound in a flipped orientation when compared to flavonoid **B4**. Thus, these flavonoids occupy the subpockets +2 to –1, with the –OH group at 7-position of A-ring in the –1 subpocket, while the B-ring binds in the other side of the active site cleft, with the B-ring facing the more hydrophobic side of the substrate cleft characterized by Val163, Leu165 and Tyr62 (+2 subpocket). The B-ring establishes aromatic stacking interactions with Tyr62 and in the case of flavonoid **C5**, the 3′–OH substitution is hydrogen bonded to the backbone of Trp58. Moving this –OH substitution to 3′-position of B-ring, as in **C4**, leads to the breakage of the hydrogen bond with Trp58 and leaves the –OH at 3′-position of B-ring more exposed to the solvent, lowering the inhibitory activity of **C4**.

The absence of an –OH group at 3-position of C-ring, as in **C6**, and the presence of an –OH substitution in the 3′ position of B-ring, define a completely different mode of interaction. In **C6**, the lack of hydrogen bond donors in the C-ring precludes the binding within the catalytic triad. Instead, B-ring binds the catalytic triad with the two –OH substitutions hydrogen bonding Asp300, while Glu233 hydrogen bonds the 3′–OH (+1 subpocket). In this binding mode, the A- and C-rings occupy the site of the substrate cleft corresponding to the –1 and –2 subpockets of the leaving maltose, with the hydrogen of the –OH at 7-position of A-ring bonding Glu240.

In what concerns D group, the binding mode of **D11**, the flavonoid with the higher inhibitory activity, is almost identical to **B4**. The C-ring carbonyl group adjoined by the –OH group of A-ring allow the binding of A- and C-rings within the catalytic triad, establishing the same hydrogen bonding network with the active site residues Asp300, His299 and Glu233 (subpocket +1). This also means that B-ring of flavonoid **D11** binds in the same site as the B-ring of flavonoid **B4** and, like **B4**, His201 hydrogen bonds the –OH group at 3′-position of B-ring, while the 3′–OH hydrogen bonds Tyr151 (subpocket –1). On the other hand, the –OH group at 7-position of A-ring seems to establish no specific interactions, while the –Cl ion at 3-position of C-ring fills the cavity formed by Ile235 and Phe256 and hydrogen bonds the backbone of Ile234 [Figure 5(B)].

A similar binding mode is predicted when the –Cl ion at 3-position of C-ring of flavonoid **D11** is substituted by an -H or –OH group (**D7** and **D8**, respectively), but the inhibitory activity is decreased by two-fold and three-fold, respectively. The lower shape complementarity of **D7** and the resistance of binding the more electronegative oxygen in the pocket formed by Ile235 and Phe256 for flavonoid **D8** might explain the decreased inhibitory activity.

It is interesting to compare **D11** with **D14**, which bears an additional –Cl substitution at 8-position of A-ring. The binding mode of **D14** is exactly the same as **D11**, thus the 8–Cl substitution binds in the hydrophobic pocket formed by Leu162, Val163 and Leu165. Despite the increased shape complementarity, the IC_50_ value increased by twofold. Perhaps a similar sized but more hydrophobic group in this position would be advantageous.

Methoxylation of the B-ring in **D16**, **D17** and **D18** flipped the mode of binding, when compared to **D11**, and disrupted the interactions with the active site triad.

Among flavonoids of the E group, it was possible to observe that exchanging the sp^2^ C2 carbon on the C-ring for a sp^3^ carbon, has a dramatic effect on the inhibitory efficiency when compared to **D5**, **D7** and **D8**. The predicted mode of binding is the same for the three flavonoids, with the A- and C-rings occupying the +3 and +2 subpockets, while the B-ring occupies the +1 subpocket and hydrogen bonds Asp197. These three flavonoids clearly cannot occupy the subpockets +1 and –1 simultaneously to maximize the interaction with the catalytic triad, thus cannot inhibit α-amylase.

## Discussion

The search for safer, specific and effective hypoglycaemic agents has been a concern of the scientific community. As a major player in the route leading to the assimilation of polysaccharides, the enzyme α-amylase plays an important role in controlling the postprandial blood glucose levels, thus being an interesting therapeutic target forT2DM. It was previously shown that the molecular models for the human and porcine pancreatic α-amylases are extremely similar^44^, which enables the use of a porcine pancreatic α-amylase surrogate in microanalysis screening of novel α-amylase inhibitors [see Supporting Information, Figure 22(S), showing the superposition of the human and porcine enzymes].

In the present work, we tested the inhibition of pancreatic α-amylase activity by a panel of 40 flavonoids, that were divided among five groups (A–E) according to the respective parent structure scaffolds, with –OH, –OMe and/or –Cl substitutions, in order to elucidate a rationale for structure-activity relationship. The kinetic analysis, inhibitory mechanism determination and the corresponding molecular docking calculations were also applied, in order to explain the binding model of the most effective flavonoid of each group and the positive control, **acarbose**, regarding the three-dimensional structure of the α-amylase catalytic site. The obtained results clearly show that α-amylase inhibition by flavonoids varies with the nature, number and position of the substituents present in the derivative flavonoids’ structure. From the studied flavonoids, the most effective compound found was the chlorinated flavone **D11**.

In what concerns the A group, the tested flavonoids (**A1**–**A6**) present low or no ability to inhibit α-amylase up to the highest tested concentration, 200 µM. Comparing the flavone **A1** with **A2**, **A3**, **A4** and **A5**, it is clear that addition of –OH groups at 3-position of C-ring and at 3′- and/or at 4′-positions of B-ring lead to no effect against the α-amylase activity. In addition, the substitution of –OH groups by –OMe groups at 3′-and 4′-positions of B-ring (**A6**) also did not lead to any effect for the inhibitory activity of α-amylase.

The most active flavonoid of B group was **B4** (IC_50_= 148 ± 5 µM). Thus, the presence of a catechol group in B-ring, together with the –OH group at 5-position of A-ring, seems distinctive features for inhibitory activity, especially if a comparison is made with those derivatives without –OH groups in B-ring (**B1**) or with just one –OH group at 3′- or 4′-positions of B-ring (**B2** and **B3**, respectively). In addition, by comparing **A5** with **B4**, it can be noticed that the –OH group seems to be more favourable at 5-position of A-ring than at 3-position of C-ring for the intended activity. This is in accordance to previous studies showing that the hydroxylation of A- (at 5- and 7-positions) and B-rings (at 3′- and 4′-positions) of flavonoids improve their inhibitory effect against this enzyme^4^ ^,^ ^45–47^.

The most effective flavonoid from the C group was **C5** (IC_50_= 59 ± 4 µM), which was tested here for the first time. Taking into account the obtained result, it is possible to conclude that the presence of an –OH group at 3-position of C-ring, at 4′-position of B-ring and at 7-position of A-ring are advantageous for the inhibitory effect. Accordingly, it was previously reported, through the study of various structurally-related flavonoids, that the hydroxylation at 4′-position of B-ring and at 7-position of A-ring in flavonoids’ scaffold is relevant for their inhibitory activity against α-amylase^4^ ^,^ ^21^ ^,^ ^45–48^. The importance of the presence of an –OH group at 4′-position in B-ring is also highlighted by comparing **C5** with **C4** (–OH group at 3′-position of the B-ring), since flavonoid **C4** presented low activity up to 200 µM, the highest tested concentration. The relevance of the presence of an –OH group at 3-position of C-ring together with an OH group at 4′-position of B-ring for the inhibition of α-amylase, is highlighted by the lack of inhibitory activity of **C3**, when compared with **C5**.

Concerning the studied methoxylated flavonoids, **C7** and **C8**, both presented no inhibitory activity against α-amylase, corroborating the idea that the inhibitory activity of flavonoids against α-amylase is not favoured through methoxyl substitution. Accordingly, it was previously described in *in vitro* studies that the methylation and methoxylation in the structure of flavonoids reduce the inhibitory activity towards α-amylase^45^ ^,^ ^46^ ^,^ ^48^.

In D group we found the most active compound of this work, flavone **D11**, with a –Cl ion at 3-position of C-ring, a –OH group at 3′ and 4′-positions of B-ring and at 5- and 7-positions of A-ring, presenting an IC_50_ value = 44 ± 3 µM. Chlorinated flavonoids were previously reported by our research group as potent anti-inflammatory agents due to their ability to suppress mechanisms engaged at the onset and progression of inflammation^27^ ^,^ ^49^. Since both oxidative stress and inflammation play a key role in the pathogenesis of insulin resistance and dysfunction of β-cells^50^, we selected these promising chlorinated flavonoids and studied their effectiveness as α-amylase inhibitors. In addition, these flavonoids already proved to be effective compounds in the inhibition of α-glucosidase^15^ and PTP1B^16^. Comparing flavones **D11** and **D8** (quercetin) (IC_50_ = 138 ± 5 µM), it is possible to conclude that the substitution of the –Cl ion by an –OH group, at 3-position of C-ring, significantly decreased the α-amylase inhibition. Moreover, the removal of the –Cl ion from 3-position of C-ring also led to a decrease of the inhibitory activity of flavonoids, as it is possible to verify by comparing **D11** with **D7** (luteolin) (IC_50_ = 78 ± 3 µM). Furthermore, the addition of an –OH group at 3-position of C-ring [**D8** (quercetin)] significantly decreased the inhibitory activity towards α-amylase (138 ± 5 µM). The comparison of flavone **D11** with **D14** show that addition of a –Cl ion at 8-position of A-ring, led to a twofold increase of the IC_50_ value (82 ± 4 µM). Thus, the presence of a –Cl ion at 3-position of the C-ring is especially advantageous for the ability of flavonoids to inhibit α-amylase activity.

Through the comparison of the results obtained for flavone **D9** (myricetin) (IC_50_ = 107 ± 6 µM) and **D8** (quercetin) (IC_50_ = 138 ± 5 µM), it was possible to observe that the addition of an –OH group at 5′-position of B-ring slightly enhanced α-amylase inhibition. The results of the present work are in accordance with those reported by Meng et al.^25^ and Tadera et al.^21^, who also studied the α-amylase inhibition by quercetin and myricetin and found that myricetin was slightly more effective than quercetin.

By comparing **D7** (luteolin) (IC_50_= 78 ± 3 µM) and **D8** (quercetin) (IC50 = 138 ± 5 µM), it was also possible to conclude that the addition of an –OH group at 3-position of C-ring decreased the flavonoids’ inhibitory activity towards α-amylase. The obtained results are in agreement with the study of Tadera et al.^21^, who also tested the mentioned flavonoids. In the study of Kim et al.^23^, the authors also observed that luteolin was an effective α-amylase inhibitor.

Comparing methoxylated flavonoids with their parent hydroxylated flavonoids, it is possible to conclude that the presence of –OMe groups decrease α-amylase inhibition, as it can be seen comparing flavone **D16** (chrysoeriol) and **D18** with **D7** (luteolin), and **D17** (acacetin) with **D5** (apigenin). These results are in agreement with previous studies found in literature^45–48^ ^,^ ^51^.

A glycosylated flavonoid was also tested, **D19** (rutin), and the presence of a sugar in the flavonoid structure did not favour its ability to inhibit α-amylase activity. In this sense, the obtained results showed that the substitution of an –OH group [**D8** (quercetin)] by a rutinose [**D19** (rutin)] at 3-position of C-ring reduced the α-amylase inhibition by flavonoids. Accordingly, it was previously described in the literature that the glycosylation of flavonoids decreases their inhibitory effect against α-amylase, possibly due to the increasing molecular size and polarity, and to the nonplanar structure^45^ ^,^ ^46^ ^,^ ^48^. In addition, this conclusion is in agreement with the study of Li et al.^24^, who tested the interaction between quercetin, rutin and isoquercetin with α-amylase, and found that the binding affinity of quercetin to the enzyme is higher when compared to rutin.

The flavanones from E group did not inhibit α-amylase, up to the highest tested concentration (200 µM). Comparing **E1** (naringenin) with **D5** (apigenin), **E2** (eriodictyol) with **D7** (luteolin) and **E3** (taxifolin) with **D8** (quercetin), it was observed that the lack of the C2 = C3 double bond did not favour the intended effect. This conclusion is in agreement with Tadera et al.,^21^ who observed that naringenin was not able to inhibit porcine pancreatic α-amylase activity, at the highest tested concentration of 0.5 mM. It is well known that the C2 = C3 double bond allows an easier entry of the molecules into the hydrophobic pockets of α-amylase since the presence of this double bond favours the near-planarity of flavonoids^4^ ^,^ ^45^ ^,^ ^46^ ^,^ ^48^.

Acarbose is a pseudotetrasaccharide produced by *Actinoplanes* sp. fermentation and its structure comprises a hydroxymethyl conduritol residue α-(1,4) linked to a 4-amino-4,6-dideoxyglucose which is, in turn, α-(1,4) linked to maltose^52^ ^,^ ^53^. It is the most widely prescribed α-glucosidase and α-amylase inhibitor. However, it was previously shown that only a mild inhibition of pancreatic α-amylase is recommended in order to avoid gastrointestinal side-effects as a result of excessive bacterial fermentation of carbohydrates in colon^11^, that has been shown as a limiting factor for T2DM treatment, in some countries^11^. In the present work, the IC_50_ values found for the tested flavonoids were higher than the IC_50_ value found for acarbose, which is 1.3 ± 0.2 µM. The mild pancreatic α-amylase inhibition prevents the abnormal bacterial fermentation of carbohydrates in the colon and consequent gastrointestinal adverse effects, such as abdominal distention, flatulence and diarrhea^11^. Moreover, it was previously shown by our research group that, in general, the tested flavonoids were more effective concerning the inhibition of α-glucosidase than the inhibition of α-amylase^15^. As such, the obtained results suggest that flavonoids could be promising α-amylase inhibitors and cause less gastrointestinal side-effects. Interestingly, it was found that the combination of baicalein or apigenin with acarbose showed additive inhibition of α-amylase at lower concentrations and antagonistic inhibition at higher concentrations. On the other hand, the combination of baicalein with acarbose synergistically inhibits α-glucosidase and lowers HGPP^54^.

After the study of α-amylase inhibition by flavonoids, we selected the most active flavonoid of each group (**B4**, **C5** and **D11**) and the positive control, **acarbose**, to test their type of inhibition.

It was described that the discrimination between mechanisms of enzymatic inhibition based on linear transformation of Michaelis–Menten equation is less accurate than the use of the nonlinear regression, since the transformation can also mask the variability inherent to kinetic data^29^ ^,^ ^30^. By the same reason, modelling on the means of repeated experiments instead of using individual data can also lead to misinterpreted mechanisms. In that sense, for the enzyme kinetic analysis, it was used the nonlinear regression to determine kinetic parameters and the type of inhibition of the most active flavonoids of each group. For this purpose, it was applied the Solver supplement of Microsoft Office Excel™. By means of iterative guesses for variables in each model equation, Solver was asked to minimize the sum of squared residuals between individual raw experimental results and the corresponding values generated by the model.

In accordance, kinetic analysis consisted in the sequential fitting of available models (without inhibition, competitive inhibition, noncompetitive inhibition, uncompetitive inhibition and mixed inhibition), and in the estimation of the kinetic constants (parameters) values. It was possible to observe by the analysis of the sum of the square errors values from the different models, and by the application of F-test and AIC test, that **acarbose** presents a mixed-type inhibition [see Supporting Information, Figures 1(S)–5(S)], and flavonoids **B4** [see Supporting Information, Figures 6(S)–10(S)], **C5** (see Supporting Information, Figures 11(S)–15(S)) and **D11** [see Supporting Information, Figures 16(S)–20(S)] present a competitive inhibition. Accordingly, Kim et al.^55^ and Yoon and Robyt,^52^ also described acarbose as a mixed type inhibitor of α-amylase. To the best of our knowledge, the inhibition type of flavonoids **B4**, **C5** and **D11** were studied here for the first time.

Once defined the best kinetic model for **acarbose** and for flavonoids **B4**, **C5** and **D11**, the kinetic constants were estimated: *V*_max_, *K*_m_, *K*_ic_ and *K*_iu_. The calculation of the Ki value is considered a useful tool to compare the potency of inhibitors. The *K*_ic_ values of the selected compounds demonstrated the following order: **B4**>**C5**>**D11**>**acarbose**. This means that, as expected, **acarbose** has the highest binding affinity to α-amylase, followed by flavonoids **D11**, **C5** and **B4**.

The prediction of the binding mode of the most active flavonoids towards α-amylase in each group showed that one of the most important aspects shared by them is a motif bearing a carbonyl group next to a –OH group. This allows the carbonyl group to accept a hydrogen bond from Glu233 and the –OH group to donate a hydrogen bond to Asp300 in the region between the –1 subpocket and +1 subpocket. The interactions with these groups can be further enhanced with a hydrogen bond between His299 and the -OH group.

The relative positioning of the -OH and carbonyl groups determine, to a great extent, the orientation of the flavonoid. Thus, the flavonoid part on the side of the carbonyl will be preferably oriented towards the +1 subpocket, while the part on the -OH side will be preferably oriented towards the –1 subpocket.

Hydrophobic groups seem to be more tolerated on the –1/–2 subpockets than on the +1/+2 subpockets, where they can interact with Tyr62, Leu162, Val163 and Leu165. Hydrophobic groups on the +1/+2 subpockets, on the other hand, can establish hydrogen bonds with Tyr151 and His201.

Halogen substitutions or hydrophobic groups might be used next to the carbonyl to explore the interaction with Ile235 and Phe256, as the best flavonoid tested herein shows.

## Conclusion

**Figure 6.**
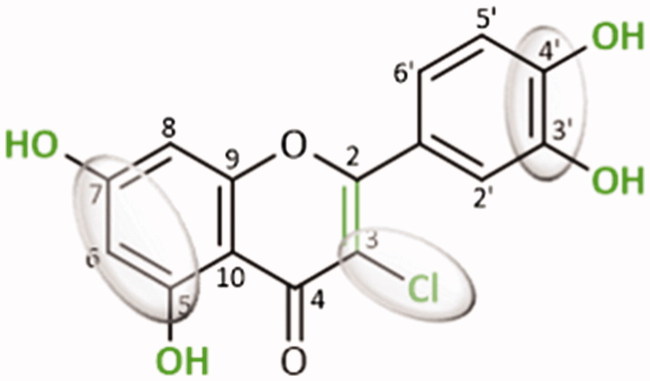
Potential substitution pattern of flavonoids contributing to the improvement of α-amylase inhibition.

In the present study, the inhibition of pancreatic α-amylase by a panel of 40 flavonoids (A–E groups) was tested, in order to establish an accurate structure-activity relationship. For that purpose, the kinetic analysis, the inhibitory mechanism determination and the corresponding molecular docking calculations were applied, to explain the binding model of the most effective flavonoid of each group to the three-dimensional structure of α-amylase catalytic site. Thus, from the obtained results, it may be concluded that α-amylase inhibition by flavonoids is strongly dependent on the nature and position of the substituents. The most effective flavonoid was the chlorinated flavone **D11**. As such, the presence of an –OH group at 5- and 7-positions of A-ring and in the 3′- and 4′-positions of B-ring, and the presence of a –Cl ion at 3-position of C-ring, as well as the C2 = C3 double bond, have shown to be favourable for the intended effect (Figure 6). This flavonoid presented a competitive type of inhibition. The promising α-amylase inhibitory activity of some of the studied flavonoids should be deeply explored allowing these compounds to be considered as possible alternatives for the management of PPHG and consequently T2DM.

## Supplementary Material

Supplemental Material
