# Supplementary material for: Evaluation of a flavonoids library for inhibition of pancreatic α-amylase towards a structure–activity relationship
Source: J Enzyme Inhib Med Chem. 2019 Feb 6;34(1):577–88. doi: 10.1080/14756366.2018.1558221 (PMC6366418; doi:10.1080/14756366.2018.1558221)
Supplement: Supplemental Material [file IENZ_A_1558221_SM6925.pdf]

Supplementary Material

Protocol details for the system preparation, energy minimization and MD simulation

The system was solvated with TIP3P water molecules, in a rectangular box whose faces were at least 12 Å away from the protein. Non-bonded Coulomb interactions were treated with the particle Mesh-Ewald method and a cutoff of 10 Å was used for explicit short-range electrostatic and Lennard-Jones interactions. All bonds involving hydrogen atoms were constrained with SHAKE algorithm and an integration step of 2 fs was employed.

The geometry optimization was done in four steps: first, only the water molecules were minimized; afterwards, the hydrogen atoms were minimized; finally, in the third step, all but the backbone was fully minimized. MD simulations started by heating the systems for 100 ps, from 0 K to 310 K, in the NVT ensemble, followed by another 500 ps of NVT MD at 310 K. Subsequently we ran 2 ns in the NPT ensemble with the protein backbone still held fixed. Temperature and pressure were maintained at 310 K and 1 bar with the Langevin thermostat and the Berendsen barostat. Finally, the constraints were removed, and a production run of 20 ns was obtained.

Enzymatic kinetic analysis

Acarbose

x values	y values	concentration of inhibitor (µM)	slopes (replicates)						standard deviation y exp
0,25	8,72	0	9,92	9,27	9,44	7,39	8,10	8,19	0,97
0,5	13,82	0	14,97	14,67	15,19	11,61	13,12	13,38	1,38
1	20,81	0	22,26	23,74	22,47	17,27	19,22	19,89	2,42
0,25	7,04	0,5	7,83	7,94	7,38	5,91	6,66	6,53	0,80
0,5	10,77	0,5	11,69	12,46	11,57	9,30	10,12	9,46	1,31
1	15,31	0,5	16,18	18,33	16,90	12,95	14,20	13,32	2,15
0,25	6,06	1	6,81	6,76	6,63	5,23	5,52	5,43	0,74
0,5	8,79	1	9,60	10,00	9,82	7,65	8,18	7,46	1,15
1	11,93	1	12,62	14,70	12,98	10,41	10,74	10,10	1,81
0,25	5,01	1,5	5,91	4,70	4,77	4,65			0,60
0,5	6,94	1,5	8,48	6,49	6,59	6,19			1,04
1	9,01	1,5	11,21	8,50	8,53	7,80			1,50
0,25	4,80	2	5,26	5,39	5,52	4,36	4,20	4,09	0,65
0,5	6,29	2	6,49	7,34	7,44	5,89	5,45	5,10	0,97
1	8,05	2	7,93	9,66	9,65	7,60	6,86	6,61	1,33

Figure 1S. Mean values of the slopes (y values) and respective standard deviations as results of the in vitro inhibition of α-amylase (0.2 U/mL) by **acarbose** (0 - 2 µM) using three concentrations of the substrate (x values: 0.25; 0.5 and 1 mM).

Without Inhib		Competitive Inhib		Noncompetitive Inhib		Uncompetitive Inhib		Mixed_Inhib	
$v_{inic} = \frac{V_{máx}(S)}{K_m + (S)}$		$v_{inic} = \frac{V_{máx}(S)}{K_m(1+\frac{\|I\|}{K_{ic}}) + (S)}$		$v_{inic} = \frac{V_{máx}(S)}{K_m(1+\frac{\|I\|}{K_{ic}}) + (S)(1+\frac{\|I\|}{K_{iu}})}$		$v_{inic} = \frac{V_{máx}(S)}{K_m + (S)(1+\frac{\|I\|}{K_{iu}})}$		$v_{inic} = \frac{V_{máx}(S)}{K_m(1+\frac{\|I\|}{K_{ic}}) + (S)(1+\frac{\|I\|}{K_{iu}})}$	
$w_i.(y_{exp} - y_{cal})^2$	Ycalc	$w_i.(y_{exp} - y_{cal})^2$	Ycalc	$w_i.(y_{exp} - y_{cal})^2$	Ycalc	$w_i.(y_{exp} - y_{cal})^2$	Ycalc	$w_i.(y_{exp} - y_{cal})^2$	Ycalc
8,3950	5,9086	0,9452	9,6587	0,3498	9,2897	0,2717	8,2114	0,0216	8,5741
14,1587	8,6308	0,1253	13,3328	0,0225	14,0282	0,0097	13,9571	0,0429	14,1068
15,6959	11,2143	3,2167	16,4644	0,6660	18,8310	0,0745	21,4679	0,0001	20,8259
1,9868	5,9086	0,0002	7,0308	0,0193	7,1532	0,0057	6,9812	0,0002	7,0314
2,6393	8,6308	0,0163	10,5986	0,0007	10,8020	0,0004	10,7402	0,0009	10,8059
3,6263	11,2143	0,2665	14,2021	0,1427	14,5002	0,0820	14,6970	0,0636	14,7703
0,0435	5,9086	0,5222	5,5270	0,1113	5,8157	0,0001	6,0716	0,0197	5,9592
0,0179	8,6308	0,0001	8,7950	0,0000	8,7823	0,0024	8,7284	0,0006	8,7569
0,1547	11,2143	0,0966	12,4865	0,0057	11,7890	0,1732	11,1731	0,0712	11,4430
2,2279	5,9086	0,5665	4,5531	0,0319	4,8996	0,3639	5,3717	0,0731	5,1707
2,6395	8,6308	0,3080	7,5159	0,1959	7,3989	0,1577	7,3514	0,1651	7,3611
2,1453	11,2143	2,0044	11,1407	0,3753	9,9319	0,0000	9,0122	0,0478	9,3392
2,8597	5,9086	2,0348	3,8710	0,7620	4,2328	0,0004	4,8164	0,1313	4,5665
5,7969	8,6308	0,0806	6,5617	0,0121	6,3920	0,0044	6,3497	0,0043	6,3490
5,6453	11,2143	2,2691	10,0567	0,1577	8,5803	0,1411	7,5517	0,0150	7,8888
68,0329	sum	12,4525	sum	2,8529	sum	1,2872	sum	0,6573	sum
V max =	16,004877	V max =	21,51844265	V max =	28,6343103	V max =	46,481198	V max =	39,76745004
K m =	0,4271901	K m =	0,306971521	K m =	0,520594918	K m =	1,1651471	K m =	0,909516227
		K ic =	0,737263624	K iu= K ic=	1,674102523	K iu =	0,5012613	K ic =	5,811649662
								K iu =	0,70959599

Figure 2S. Sum of the squares (sum) of the different models (without inhibition, competitive inhibition, noncompetitive inhibition, uncompetitive inhibition and mixed inhibition) from the results obtained from α-amylase inhibition by **acarbose**.

Comparison based on F test

	$w_i \cdot (y_{exp} - y_{cal})^2$	p	n	W Without _Inhib	n-pB	$f_{0,05}$ ν-f)	Without _Inhib)	Uncompetitive Inhib	
Without Inhib	68,0329	2	15						
Competitive Inhib	12,4525	3	15	53,5605	12	4,8	48,81		
Noncompetitive Inhib	2,8529	3	15	274,1656	12	4,8	269,42		
Uncompetitive Inhib	1,2872	3	15	622,2598	12	4,8	617,51		se ↓ > 0 prefer Mixed Inhib
Mixed Inhib	0,6573	4	15	563,7400	11	4	559,76	6,56	

Comparison based on Akaike test

	AIC c	ΔAICc			
Without Inhib	30,5253				
Competitive Inhib	8,5415	-21,98			
Noncompetitive Inhib	-13,5625	-44,09			
Uncompetitive Inhib	-25,5008	-56,03			
Mixed Inhib	-31,4596	-61,98	-40,00	-17,90	-5,96

Figure 3S. Comparison of the different models (without inhibition, competitive inhibition, noncompetitive inhibition, uncompetitive inhibition and mixed inhibition) applying the F test and the Akaike test, obtained from α-amylase inhibition by **acarbose**.

Calculation of parameters errors by the "jacknife" procedure

	V max	K m	K ic	K iu
1	42,063	1,006	7,687	0,651
2	40,159	0,909	5,498	0,698
3	40,041	0,918	5,896	0,702
4	39,817	0,912	5,824	0,709
5	39,784	0,910	5,801	0,710
6	38,810	0,879	5,827	0,723
7	39,908	0,915	5,565	0,712
8	39,765	0,910	5,815	0,709
9	39,530	0,902	6,260	0,695
10	40,043	0,921	7,553	0,681
11	39,712	0,909	5,854	0,724
12	39,580	0,904	5,279	0,739
13	38,999	0,877	4,290	0,761
14	39,750	0,910	5,827	0,712
15	40,031	0,918	6,263	0,688

Error of V max:	2,578
Error of K m:	0,104
Error of K ic:	2,967
Error of K iu:	0,092

Figure 4S. Determination of the errors of the parameters (Vmax, Km, Kic and Kiu) for the mixed inhibition model of α-amylase by **acarbose**, using the jackknife procedure.

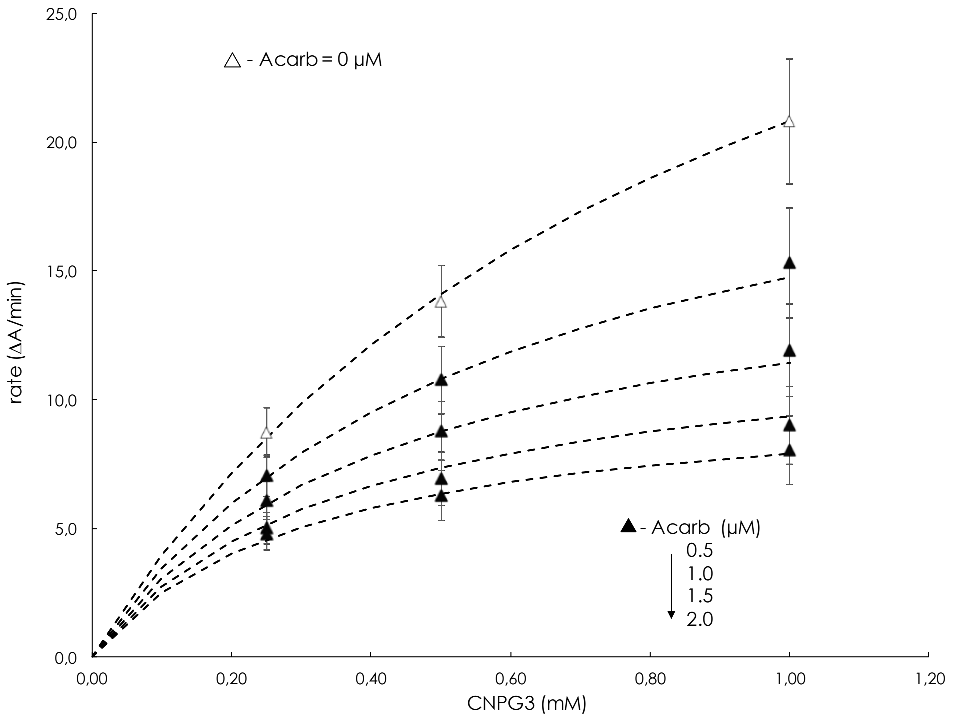

Figure 5S. Michaelis-Menten representation of the mixed inhibition model of **acarbose**.

Flavonoid B4

Nonlinear regression using SOLVER

x values	y values	concentration of inhibitor	slopes (replicates)						standard deviation y exp
0,25	7,31	0	7,82	7,87	6,46	7,08			0,67
0,5	11,89	0	12,18	12,06	11,68	11,65			0,27
1	18,32	0	18,42	18,40	17,80	18,68			0,37
0,25	6,26	25	6,81	6,74	5,50	5,99			0,63
0,5	10,53	25	11,15	10,77	10,25	9,94			0,54
1	16,84	25	17,36	16,86	16,48	16,66			0,38
0,25	5,68	50	6,17	6,10	5,05	5,42			0,54
0,5	9,58	50	10,29	10,05	9,25	8,75			0,71
1	15,55	50	16,06	15,84	15,38	14,92			0,51
0,25	4,66	100	5,02	4,95	4,24	4,43			0,38
0,5	7,93	100	8,43	8,19	7,57	7,52			0,45
1	13,44	100	14,09	13,75	12,65	13,28			0,62
0,25	3,21	200	3,62	3,53	2,57	3,11			0,48
0,5	5,41	200	5,62	5,70	5,21	5,13			0,29
1	10,45	200	10,88	10,13	9,46	11,31			0,82

Figure 6S. Mean values of the slopes (y values) and respective standard deviations as results of the in vitro inhibition of α-amylase (0.2 U/mL) by flavonoid **B4** (0 - 200 µM) using three concentrations of the substrate (x values: 0.25; 0.5 and 1 mM).

Without Inhib

$$v_{inic} = \frac{V_{máx}(S)}{K_m + (S)}$$

Competitive Inhib

$$v_{inic} = \frac{V_{máx}(S)}{K_m(1 + \frac{|I|}{K_{ic}}) + (S)}$$

Noncompetitive Inhib

$$v_{inic} = \frac{V_{máx}(S)}{K_m(1 + \frac{|I|}{K_{ic}}) + (S)(1 + \frac{|I|}{K_{iu}})}$$

Uncompetitive Inhib

$$v_{inic} = \frac{V_{máx}(S)}{K_m + (S)(1 + \frac{|I|}{K_{iu}})}$$

Mixed Inhib

$$v_{inic} = \frac{V_{máx}(S)}{K_m(1 + \frac{|I|}{K_{ic}}) + (S)(1 + \frac{|I|}{K_{iu}})}$$

Without Inhib: $w_i.(y_{exp} - y_{cal})^2$	Without Inhib: Ycalc	Competitive Inhib: $w_i.(y_{exp} - y_{cal})^2$	Competitive Inhib: Ycalc	Noncompetitive Inhib: $w_i.(y_{exp} - y_{cal})^2$	Noncompetitive Inhib: Ycalc	Uncompetitive Inhib: $w_i.(y_{exp} - y_{cal})^2$	Uncompetitive Inhib: Ycalc	Mixed Inhib: $w_i.(y_{exp} - y_{cal})^2$	Mixed Inhib: Ycalc
21,1224	4,2150	0,1416	7,0522	0,6418	6,7666	2,3393	6,2768	0,1416	7,0522
164,8397	8,4299	0,1332	11,9917	0,1258	11,7976	3,5906	11,3821	0,1332	11,9917
15,5156	16,8598	0,1233	18,4545	1,4961	18,7787	5,3487	19,1837	0,1234	18,4546
10,5990	4,2150	0,0604	6,1031	0,1232	6,0371	0,2113	5,9689	0,0604	6,1031
15,3437	8,4299	0,0136	10,5912	0,0000	10,5258	0,0504	10,4084	0,0136	10,5912
0,0030	16,8598	0,0543	16,7502	0,0494	16,7543	0,4947	16,5710	0,0542	16,7503
7,3762	4,2150	0,3182	5,3791	0,1881	5,4496	0,0001	5,6898	0,3182	5,3791
2,6469	8,4299	0,0203	9,4836	0,0138	9,5015	0,0000	9,5882	0,0203	9,4837
6,6473	16,8598	0,1806	15,3341	0,7036	15,1239	3,6112	14,5846	0,1804	15,3342
1,3369	4,2150	0,6585	4,3476	0,0649	4,5618	1,9951	5,2031	0,6584	4,3476
1,2409	8,4299	0,0351	7,8432	0,0033	7,9535	0,6202	8,2827	0,0351	7,8433
29,9973	16,8598	0,2669	13,1163	1,5559	12,6599	7,1900	11,7642	0,2668	13,1164
4,4464	4,2150	0,0171	3,1424	0,2421	3,4407	6,6823	4,4431	0,0171	3,1424
110,2694	8,4299	2,0689	5,8273	4,1443	5,9989	14,5598	6,5101	2,0694	5,8274
61,5944	16,8598	0,1103	10,1735	1,2026	9,5486	5,7604	8,4833	0,1103	10,1736
452,9791	sum	4,2022	sum	10,5549	sum	52,4541	sum	4,2023	sum

Without Inhib		Competitive Inhib		Noncompetitive Inhib		Uncompetitive Inhib		Mixed Inhib	
V max =	1,48E+12	V max =	40,026555	V max =	45,99664125	V max =	60,982914	V max =	40,027209
K m =	8,777E+10	K m =	1,1689294	K m =	1,449410179	K m =	2,1788956	K m =	1,1689555
		K ic =	132,42223	K iu= K ic=	206,904099	K iu =	49,878935	K ic =	132,42602
								K iu =	9461939,8

Figure 7S. Sum of the squares (sum) of the different models (without inhibition, competitive inhibition, noncompetitive inhibition, uncompetitive inhibition and mixed inhibition) from the results obtained from α-amylase inhibition by flavonoid **B4**.

Comparison based on F test:

	$w_i \cdot (y_{exp} - y_{cal})^2$	p	n	W Without Inhib	n-pB	$f_{0,05}$	ν-f) Without Inhib
Without Inhib	452,9791	2	15				
Competitive Inhib	4,2022	3	15	1281,5356	12	4,75	1276,79
Noncompetitive Inhib	10,5549	3	15	502,9980	12	4,75	498,25
Uncompetitive Inhib	52,4541	3	15	91,6286	12	4,75	86,88
Mixed Inhib	4,2023	4	15	587,3616	11	3,98	583,38

Comparison based on Akaike test:

	AIC c	ΔAICc			
Without Inhib	58,9631				
Competitive Inhib	-7,7531	-66,72			
Noncompetitve Inhib	6,0614	-52,90			
Uncompetitive Inhib	30,1117	-28,85			
Mixed Inhib	-3,6317	-62,59	4,12	-9,69	-33,74

Figure 8S. Comparison of the different models (without inhibition, competitive inhibition, noncompetitive inhibition, uncompetitive inhibition and mixed inhibition) applying the F test and the Akaike test, obtained from α-amylase inhibition by flavonoid **B4.**

Calculation of the error parameters by the "jacknife"procedure

	V max	K m	K ic	K iu
1	40,411	1,189	133,757	
2	38,390	1,074	124,109	
3	41,271	1,217	132,044	
4	40,213	1,178	132,864	
5	39,948	1,165	132,221	
6	39,597	1,151	131,667	
7	40,412	1,188	132,888	
8	40,077	1,172	132,428	
9	39,620	1,151	130,897	
10	40,543	1,193	131,839	
11	40,103	1,172	132,022	
12	39,732	1,155	130,211	
13	40,058	1,170	132,227	
14	39,338	1,146	145,187	
15	39,935	1,164	131,125	

Error of V max:	2,311
Error of K m:	0,114
Error of K ic:	15,103
Error of K iu:	#DIV/0!

Figure 9S. Determination of the errors of the parameters (Vmax, Km and Kic) for the competitive inhibition model of α-amylase by flavonoid **B4**, using the jackknife procedure.

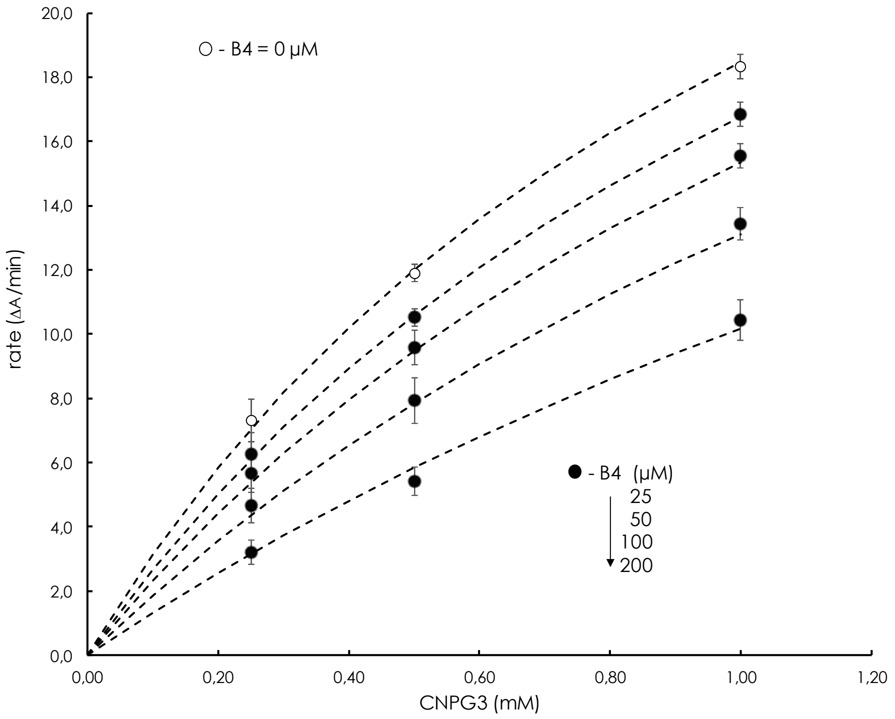

Figure 10S. Michaelis-Menten representation of the competitive inhibition model of flavonoid **B4**.

Flavonoid C5

Nonlinear regression using SOLVER

x values	y values	Concentrations of inhibitor	slopes (replicates)						standard deviation y exp
0,25	7,36	0	7,004	7,598	7,47				0,31
0,5	11,56	0	11,842	11,378	11,464				0,25
1	17,73	0	18,512	16,837	17,837				0,84
0,25	6,39	12,5	6,15	6,7	6,313				0,28
0,5	10,35	12,5	10,474	10,23	10,349				0,12
1	16,52	12,5	16,21	16,53	16,816				0,30
0,25	5,80	25	5,749	5,97	5,679				0,15
0,5	9,38	25	8,979	9,72	9,455				0,38
1	15,37	25	14,696	15,75	15,678				0,59
0,25	4,65	50	4,498	4,78	4,685				0,14
0,5	7,87	50	7,617	8,34	7,655				0,41
1	13,34	50	12,856	13,75	13,401				0,45
0,25	3,30	100	3,186	3,52	3,196				0,19
0,5	5,80	100	5,69	6,02	5,684				0,19
1	10,66	100	10,025	11,23	10,717				0,60

Figure 11S. Mean values of the slopes (y values) and respective standard deviations as results of the in vitro inhibition of α-amylase (0.2 U/mL) by flavonoid **C5** (0 - 100 µM) using three concentrations of the substrate (x values: 0.25; 0.5 and 1 mM).

Without Inhib

$$v_{inic} = \frac{V_{máx}(S)}{K_m + (S)}$$

Competitive Inhib

$$v_{inic} = \frac{V_{máx}(S)}{K_m(1 + \frac{|I|}{K_{ic}}) + (S)}$$

Noncompetitive Inhib

$$v_{inic} = \frac{V_{máx}(S)}{K_m(1+\frac{|I|}{K_{ic}}) + (S)(1+\frac{|I|}{K_{iu}})}$$

Uncompetitive Inhib

$$v_{inic} = \frac{V_{máx}(S)}{K_m + (S)(1+\frac{|I|}{K_{iu}})}$$

Mixed Inhib

$$v_{inic} = \frac{V_{máx}(S)}{K_m(1+\frac{|I|}{K_{ic}}) + (S)(1+\frac{|I|}{K_{iu}})}$$

Without Inhib $w_i.(y_{exp} - y_{cal})^2$	Without Inhib Ycalc	Competitive Inhib $w_i.(y_{exp} - y_{cal})^2$	Competitive Inhib Ycalc	Noncompetitive Inhib $w_i.(y_{exp} - y_{cal})^2$	Noncompetitive Inhib Ycalc	Uncompetitive Inhib $w_i.(y_{exp} - y_{cal})^2$	Uncompetitive Inhib Ycalc	Mixed Inhib $w_i.(y_{exp} - y_{cal})^2$	Mixed Inhib Ycalc
48,5374	5,1794	0,9144	7,0584	3,4022	6,7807	14,1545	6,1812	0,9143	7,0584
88,4926	9,2393	0,6355	####	0,3428	11,7059	0,2960	11,4270	0,6352	11,7581
9,0418	15,1946	0,0148	####	0,6002	18,3816	6,3382	19,8503	0,0149	17,6259
18,2941	5,1794	0,4808	6,1918	1,1812	6,0806	3,6260	5,8497	0,4807	6,1918
83,0157	9,2393	2,1638	####	1,4378	10,4973	0,0037	10,3435	2,1636	10,5305
19,0761	15,1946	1,0385	####	0,0132	16,4838	0,8267	16,7943	1,0393	16,2096
16,6602	5,1794	3,5122	5,5147	3,5886	5,5116	2,6515	5,5520	3,5113	5,5147
0,1499	9,2393	0,1602	9,5350	0,1204	9,5149	0,0282	9,4477	0,1603	9,5350
0,0935	15,1946	0,3962	####	0,5419	14,9412	1,9437	14,5537	0,3963	15,0040
13,3905	5,1794	0,8122	4,5250	0,0066	4,6427	7,1905	5,0391	0,8117	4,5251
11,3138	9,2393	0,1326	8,0188	0,1256	8,0148	0,2005	8,0528	0,1326	8,0189
17,0217	15,1946	0,3715	####	2,7712	12,5856	16,8105	11,4883	0,3715	13,0610
97,7562	5,1794	0,0236	3,3299	1,4528	3,5297	25,1298	4,2532	0,0237	3,3299
320,3130	9,2393	2,2123	6,0840	2,3616	6,0935	4,7499	6,2171	2,2131	6,0840
56,2974	15,1946	0,2192	####	3,2419	9,5685	18,1199	8,0832	0,2192	10,3742
799,4540	sum	13,0877	sum	21,1881	sum	102,0697	sum	13,0877	sum

Without Inhib		Competitive Inhib		Noncompetitive Inhib		Uncompetitive Inhib		Mixed Inhib	
V max =	42,748451	V max =	35,186	V max =	42,776464	V max =	75,515731	V max =	35,185132
K m =	1,8134007	K m =	0,9963	K m =	1,32713844	K m =	2,8042553	K m =	0,9962122
		K ic =	71,393	K iu= K ic=	108,572306	K iu =	18,056995	K ic =	71,39385
								K iu =	35359966

Figure 12S. Sum of the squares (sum) of the different models (without inhibition, competitive inhibition, noncompetitive inhibition, uncompetitive inhibition and mixed inhibition) from the results obtained from α-amylase inhibition by flavonoid **C5**.

Comparison based on F test

	$w_i \cdot (y_{exp} - y_{cal})^2$	p	n	W Without Inhib	n-pB	$f_{0,05}$	v-f) Without Inhib	(W-f) Mixed Inhib
Without Inhib	799,4540	2	15					
Competitive Inhib	13,0877	3	15	721,0112	12	4,75	716,26	
Noncompetitive Inhib	21,1881	3	15	440,7758	12	4,75	436,03	
Uncompetitive Inhib	102,0697	3	15	81,9892	12	4,75	77,24	
Mixed Inhib	13,0877	4	15	330,4633	11	3,98	326,48	-3,98

Comparison based on Akaike test

	AIC c	ΔAICc			
Without Inhib	67,4843				
Competitive Inhib	9,2877	-58,20			
Noncompetitive Inhib	16,5142	-50,97			
Uncompetitive Inhib	40,0974	-27,39			
Mixed Inhib	13,4089	-54,08	4,12	-3,11	-26,69

Figure 13S. Comparison of the different models (without inhibition, competitive inhibition, noncompetitive inhibition, uncompetitive inhibition and mixed inhibition) applying the F test and the Akaike test, obtained from α-amylase inhibition by flavonoid **C5.**

Claculation of parameters errors based on "jacknife" procedure

	V max	K m	K ic	K iu
1	35,852	1,031	73,289	
2	35,060	0,981	69,359	
3	35,107	0,993	71,377	
4	35,555	1,014	72,071	
5	34,831	0,957	68,194	
6	32,522	0,890	69,179	
7	36,891	1,076	72,650	
8	35,174	0,995	71,441	
9	34,772	0,979	70,823	
10	35,728	1,018	70,581	
11	35,180	0,996	71,650	
12	34,753	0,977	70,294	
13	35,155	0,995	71,607	
14	35,254	1,010	77,253	
15	35,029	0,988	70,665	

Error of V max:	3,256
Error of K m:	0,144
Error of Kic:	7,611
Error of Kiu:	#DIV/0!

Figure 14S. Determination of the errors of the parameters (Vmax, Km and Kic) for the competitive inhibition model of α-amylase by flavonoid **C5**, using the jackknife procedure.

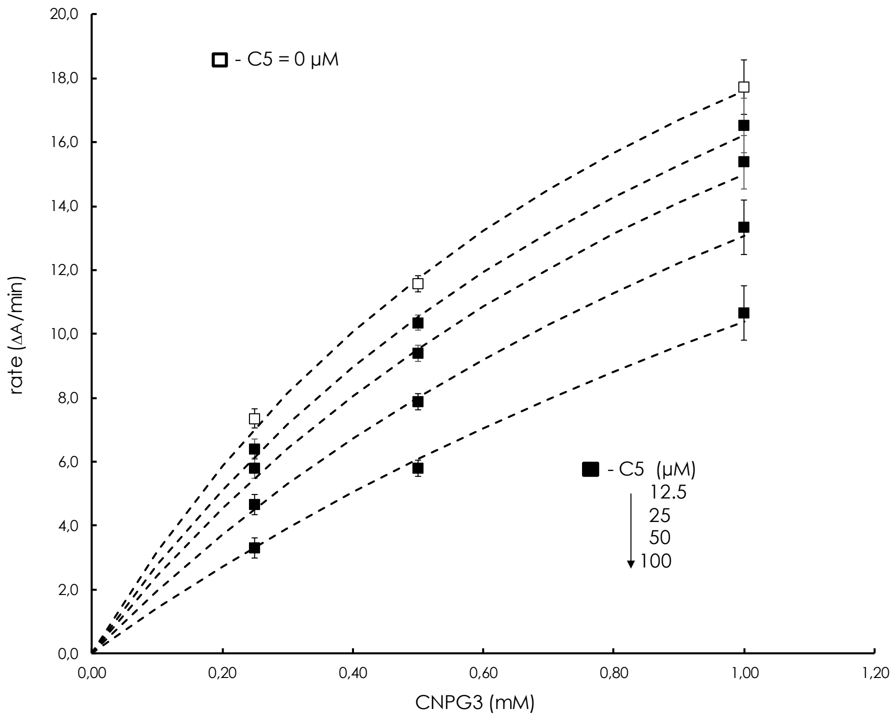

Figure 15S. Michaelis-Menten representation of the competitive inhibition model of flavonoid **C5**.

Flavonoid D11

Nonlinear regression using SOLVER

x values	y values	concentrations of inhibitor	slopes (replicates)						standard deviation y exp
0,25	8,58	0	8,25	7,53	9,59	8,94			0,89
0,5	13,90	0	13,10	12,53	15,16	14,82			1,28
1	21,77	0	20,32	19,74	23,70	23,34			2,03
0,25	6,87	12,5	6,35	6,02	8,01	7,09			0,88
0,5	11,45	12,5	10,85	9,62	12,74	12,58			1,49
1	18,58	12,5	17,30	16,67	19,94	20,40			1,87
0,25	5,36	25	4,89	4,30	6,38	5,85			0,93
0,5	9,19	25	8,48	8,02	10,07	10,18			1,10
1	16,19	25	14,67	14,47	17,94	17,69			1,88
0,25	3,54	50	3,40	2,80	4,17	3,81			0,59
0,5	6,29	50	5,84	5,39	6,97	6,94			0,79
1	11,68	50	10,82	10,31	12,90	12,68			1,30
0,25	1,75	100	1,79	1,44	1,92	1,85			0,21
0,5	3,29	100	2,89	3,28	3,38	3,59			0,29
1	6,45	100	6,15	6,38	6,43	6,84			0,29

Figure 16S. Mean values of the slopes (y values) and respective standard deviations as results of the in vitro inhibition of α-amylase (0.2 U/mL) by flavonoid **D11** (0 - 100 μM) using three concentrations of the substrate (x values: 0.25; 0.5 and 1 mM).

Without Inhib

$$v_{inic} = \frac{V_{máx}(S)}{K_m + (S)}$$

Competitive Inhib

$$v_{inic} = \frac{V_{máx}(S)}{K_m(1 + \frac{|I|}{K_{ic}}) + (S)}$$

Noncompetitive Inhib

$$v_{inic} = \frac{V_{máx}(S)}{K_m(1+\frac{|I|}{K_{ic}}) + (S)(1+\frac{|I|}{K_{iu}})}$$

Uncompetitive Inhib

$$v_{inic} = \frac{V_{máx}(S)}{K_m + (S)(1+\frac{|I|}{K_{iu}})}$$

Mixed Inhib

$$v_{inic} = \frac{V_{máx}(S)}{K_m(1+\frac{|I|}{K_{ic}}) + (S)(1+\frac{|I|}{K_{iu}})}$$

Without Inhib $w_i.(y_{exp} - y_{cal})^2$	Ycalc	Competitive Inhib $w_i.(y_{exp} - y_{cal})^2$	Ycalc	Noncompetitive Inhib $w_i.(y_{exp} - y_{cal})^2$	Ycalc	Uncompetitive Inhib $w_i.(y_{exp} - y_{cal})^2$	Ycalc	Mixed Inhib $w_i.(y_{exp} - y_{cal})^2$	Ycalc
54,7655	2,0117	0,1539	8,9253	0,4855	7,9591	8,3971	6,0064	0,1067	8,8670
59,2554	4,0233	0,7936	15,0435	0,2718	14,5694	2,1650	12,0125	0,7706	15,0268
45,5709	8,0467	0,3008	22,8886	2,3893	24,9163	1,2255	24,0242	0,3785	23,0242
30,3184	2,0117	0,8844	6,0390	1,3695	5,8363	2,7608	5,4029	0,8537	6,0535
24,8965	4,0233	0,2367	10,7240	0,2640	10,6835	1,1983	9,8191	0,2218	10,7473
31,8415	8,0467	0,3210	17,5201	0,0270	18,2707	1,1164	16,6056	0,3020	17,5520
12,7996	2,0117	0,7185	4,5633	0,6407	4,6074	0,2276	4,9096	0,6614	4,5954
22,0065	4,0233	0,6035	8,3317	0,4677	8,4340	0,6446	8,3030	0,5574	8,3650
18,8419	8,0467	1,1379	14,1915	0,8893	14,4236	3,4891	12,6877	1,1493	14,1815
6,7784	2,0117	0,6627	3,0652	0,2638	3,2421	1,0631	4,1516	0,5666	3,1013
8,1163	4,0233	0,4362	5,7613	0,1954	5,9347	0,0054	6,3440	0,3810	5,7956
7,7577	8,0467	1,1434	10,2839	1,3744	10,1495	5,5011	8,6201	1,2059	10,2463
1,5197	2,0117	0,2208	1,8503	1,8122	2,0356	45,1633	3,1720	0,3683	1,8793
6,3364	4,0233	0,8975	3,5629	2,2638	3,7263	12,2142	4,3101	1,0821	3,5901
30,9061	8,0467	0,4015	6,6318	0,0720	6,3727	17,3765	5,2523	0,2361	6,5893
361,7108	sum	8,9123	sum	12,7864	sum	102,5481	sum	8,8413	sum

Without Inhib		Competitive Inhib		Noncompetitive Inhib		Uncompetitive Inhib		Mixed Inhib	
V max =	8,64E+08	V max =	47,8328	V max =	85,972991	V max =	364404,77	V max =	49,21848
K m =	1,07E+08	K m =	1,08981	K m =	2,4504661	K m =	15167,213	K m =	1,1376846
		K ic =	21,2735	K iu= K ic=	34,365917	K iu =	0,0018446	K ic =	22,297304
								K iu =	435,9238

Figure 17S. Sum of the squares (sum) of the different models (without inhibition, competitive inhibition, noncompetitive inhibition, uncompetitive inhibition and mixed inhibition) from the results obtained from α-amylase inhibition by flavonoid **D11**.

Comparison based on F test

	$w_i \cdot (y_{exp} - y_{cal})^2$	p	n	W Without Inhib	n-pB	$f_{0,05}$	'it hout inhib/Mixed inhib	
Without Inhib	361,7108	2	15					
Competitive Inhib	8,9123	3	15	475,0266	12	4,75	470,28	
Noncompetitive Inhib	12,7864	3	15	327,4656	12	4,75	322,72	
Uncompetitive Inhib	102,5481	3	15	30,3268	12	4,75	25,58	
Mixed Inhib	8,8413	4	15	219,5141	11	3,98	215,53	-3,89

Comparison based on Akaike test

	AIC c	ΔAICc			
Without Inhib	55,5881				
Competitive Inhib	3,5241	-52,06			
Noncompetitive Inhib	8,9383	-46,65			
Uncompetitive Inhib	40,1676	-15,42			
Mixed Inhib	7,5252	-48,06	4,00	-1,41	-32,64

Figure 18S. Comparison of the different models (without inhibition, competitive inhibition, noncompetitive inhibition, uncompetitive inhibition and mixed inhibition) applying the F test and the Akaike test, obtained from α-amylase inhibition by flavonoid **D11.**

Calculation of the error parameters by the "jacknife" procedure

	V max	K m	K ic	K iu
1	43,481	0,912	19,501	
2	47,592	1,032	19,979	
3	59,298	1,428	22,019	
4	51,086	1,217	22,171	
5	48,067	1,105	21,463	
6	45,016	1,006	20,938	
7	49,400	1,149	21,617	
8	48,357	1,115	21,450	
9	45,030	1,005	20,830	
10	49,127	1,135	21,435	
11	48,366	1,110	21,294	
12	46,099	1,035	20,825	
13	46,731	1,054	21,260	
14	46,075	1,035	21,504	
15	50,645	1,194	23,083	

Error of V max:	13,443
Error of K m:	0,435
Error of K ic:	3,089
Error of K iu:	#DIV/0!

Figure 19S. Determination of the errors of the parameters (Vmax, Km and Kic) for the competitive inhibition model of α-amylase by flavonoid **D11**, using the jackknife procedure.

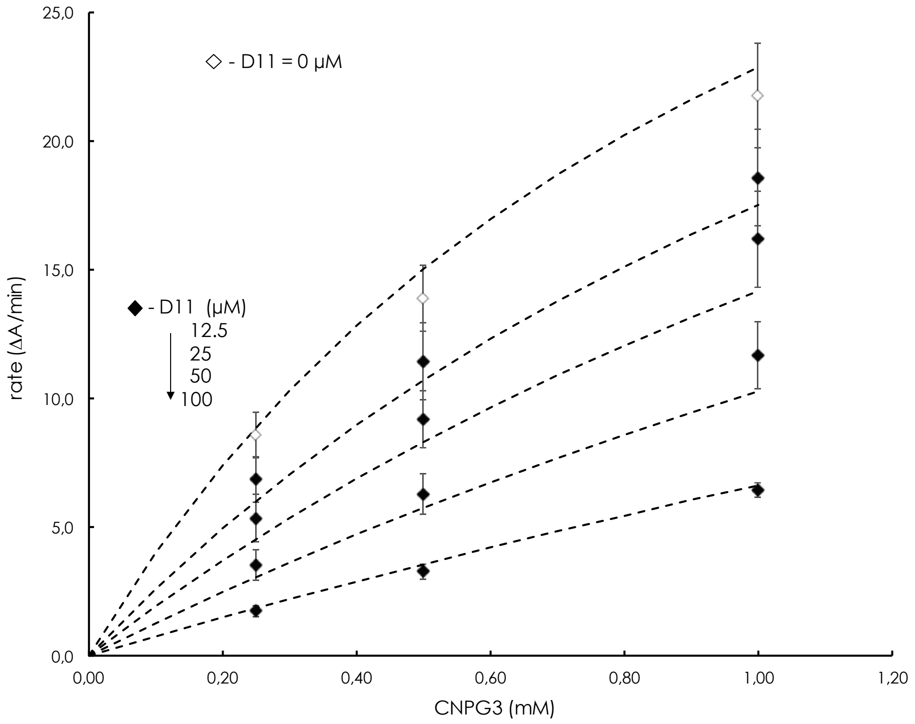

Figure 20S. Michaelis-Menten representation of the competitive inhibition model of flavonoid **D11**.

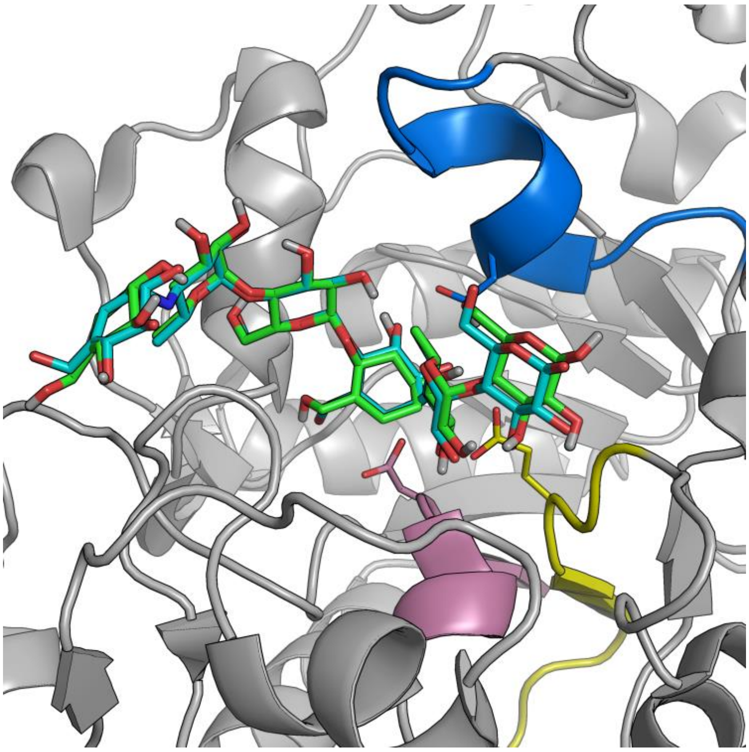

Figure 21S. Molecular docking assay used to confirm the predictive power of the protocol. Detailed picture of the active site region of alpha-amylase crystallographic structure (retrieved from PDB Entry: 1UA7), displaying the original acarbose-derived hexasaccharide present in the crystallographic structure (in cyan) and the re-docked acarbose-derived hexasaccharide (in green). Active site residues Asp176, Glu208 and Asp269 are also depicted in yellow, blue and pink, respectively.

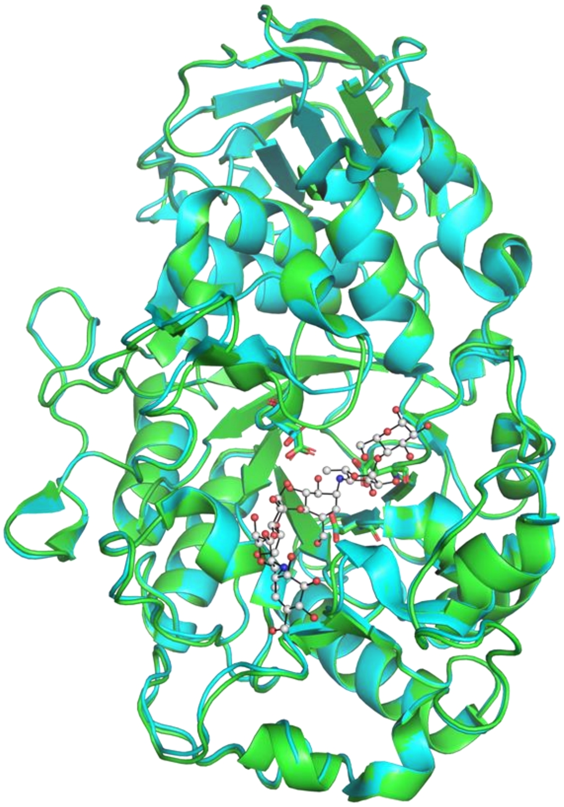

Figure 22S. Structural comparison of porcine pancreatic α-amylase (PDB:1HX0, green) and human pancreatic α-amylase (PDB: 5U3A, cyan). In the overlay, the active site triad of both structures is represented in sticks and the trisaccharide inhibitor, K2, from PDB 1HX0 is also represented.
